# Efficacy of the mHealth-Based Exercise Intervention re.flex for Patients With Knee Osteoarthritis: Pilot Randomized Controlled Trial

**DOI:** 10.2196/54356

**Published:** 2024-09-09

**Authors:** Valerie Dieter, Pia Janssen, Inga Krauss

**Affiliations:** 1 Department of Sports Medicine Medical Clinic University Hospital Tübingen Tübingen Germany; 2 Interfaculty Research Institute for Sports and Physical Activity Tübingen Germany

**Keywords:** digital app, mobile health, mHealth, knee osteoarthritis, exercise, knee brace

## Abstract

**Background:**

Exercise therapy is recommended by international guidelines as a core treatment for patients with knee osteoarthritis. However, there is a significant gap between recommendations and practice in health care. Digital exercise apps are promising to help solve this undersupply.

**Objective:**

This study aims to evaluate the efficacy of a 12-week fully automated app-based exercise intervention with and without a supporting knee brace on health-related outcomes, performance measures, and adherence in patients with knee osteoarthritis.

**Methods:**

This closed user group trial included participants with moderate to severe unicondylar painful knee osteoarthritis. Randomization was 1:1:2 into an intervention group (IG) with 2 subgroups (app-based training [IG A] and app-based training and a supportive knee brace [IG AB]) and a control group (CG). The intervention included a 12-week home exercise program with 3 sessions per week. Instructions for the exercises were given via the app and monitored using 2 accelerometers placed below and above the affected knee joint. Participants in the CG did not receive any study intervention but were allowed to make use of usual care. Osteoarthritis-specific pain (Knee Injury and Osteoarthritis Outcome Score) was defined as the primary outcome, and secondary outcomes included all other Knee Injury and Osteoarthritis Outcome Score subscales, general health-related quality of life (Veterans RAND 12-item Health Survey), psychological measures (eg, exercise self-efficacy), performance measures (strength and postural control), and the monitoring of adherence and safety. Outcomes were assessed at baseline and after 12 weeks. Intervention effects were calculated using baseline-adjusted analysis of covariance for the joint comparison of IG A and IG AB versus the CG using a per-protocol approach. Subgroup analyses were conducted for each IG separately.

**Results:**

A total of 61 participants were included (IG: n=30, 49%; CG: n=31, 51%; male: n=31, 51%; female: n=30, 49%; mean age 62.9, SD 8.5 years; mean BMI 27.7, SD 4.5 kg/m^2^). Analysis revealed statistically significant effects in favor of the IG for pain reduction (*P*<.001; effect size [ES]=0.76), improvements in physical function (*P*<.001; ES=0.64), improvements in symptoms (*P*=.01; ES=0.53), improvements in sport and recreation activities (*P*=.02; ES=0.47), improvements in knee-related quality of life (*P*<.001; ES=0.76), and improvements in the physical component of general health-related quality of life (*P*<.001; ES=0.74). Mean differences ranged from 6.0 to 13.2 points (scale range 0-100). ESs indicated small to medium effects. No effects were found for psychological and performance measures. Participants adhered to 92.5% (899/972) of all scheduled exercise sessions.

**Conclusions:**

Individuals with knee osteoarthritis undergoing a 12-week sensor-assisted app-based exercise intervention with or without an additional knee brace experienced clinically meaningful treatment effects regarding pain relief and improvements in physical function as well as other osteoarthritis-specific concerns compared to controls.

**Trial Registration:**

German Clinical Trials Register (DRKS) DRKS00023269; https://drks.de/search/de/trial/DRKS00023269

## Introduction

### Background

Osteoarthritis is a degenerative joint disease and one of the major contributors to global disabilities [1]. The prevalence rate of osteoarthritis increases with age, with women being more frequently affected than men [1,2]. Almost 30% of the German population in the sixth decade of life have been diagnosed with osteoarthritis [3]. The knee joint is the most commonly affected joint of the lower extremities. With disease progression, knee osteoarthritis is more frequently associated with increasing pain, limitations in physical function [4], and decreased health-related quality of life (HRQoL). National and international guidelines recommend exercise therapy as a nonpharmacological core treatment for patients with knee osteoarthritis [5-8]. Exercise programs have shown to decrease pain and improve physical function [9]. They can include strengthening exercises but also aerobic training, neuromuscular training, balance training, mixed exercise programs, aquatic exercises, or mind-body activities such as tai chi or yoga [5,6,8,10]. Different training settings (individual, group based, and home based) have also shown to be effective, allowing patients to exercise according to their individual preferences [5]. However, it is suggested that most people with knee osteoarthritis need some form of ongoing monitoring or supervision to optimize the clinical benefits of exercise treatment [9,10]. Despite given consensus on the need to recommend exercise with some kind of supervision, there is a considerable discrepancy regarding its implementation in health care. In 2016, <40% of patients with hip, knee, or polyarticular osteoarthritis who were customers of a German statutory health insurance company received a prescription for therapeutic exercise [11], and similar numbers have been described in an international meta-analysis [12]. Therefore, it seems reasonable to explore alternative approaches for people with limited access to therapeutic services [13]. In this regard, digital apps for exercise instructions could be particularly suitable to support patients in doing exercises. A recently conducted questionnaire study with health care professionals revealed very high acceptance of mobile health (mHealth)–based intervention therapies in osteoarthritis treatment. This indicates that they would also recommend or prescribe m-Health exercise interventions [14]. The main advantages of apps are related to their use independent of time and location, making this kind of intervention available for many patients even in rural areas [15-17]. In addition, special app features such as information and advice for guidance, tracking and self-monitoring of health behavior, feedback mechanisms, and reminders via push notifications can be of particular value [18,19]. The integration of accelerometers can additionally support patients in conducting exercises in a correct and safe manner by imitating human supervision. In general, 2 main types of mHealth apps are differentiated: interactive and stand-alone apps. Interactive mHealth apps can be used for communication between patients and health care professionals such as physical therapists [20]. These kinds of apps are frequently used in the context of blended care [21]. In contrast, stand-alone apps do not involve interaction with a health care professional, and patients exercise autonomously [22,23].

Both kinds of apps can provide an added value for patients by supporting them in implementing and maintaining exercise in their life and profiting from associated health benefits. A recent meta-analysis reported short-term improvements in pain relief and quality of life (QoL) in patients with knee osteoarthritis or chronic knee pain following the use of technology-based exercise and physical activity programs [19]. However, only 2 of 12 included randomized controlled trials explicitly used an mHealth app [23,24], and only one of these examined a structured exercise program [23]. Therefore, it seems reasonable to conduct further research specifically to evaluate mHealth-based structured exercise programs for patients with knee osteoarthritis. Consideration should also be given to the type of interaction between patients and health care providers in the app.

In addition to exercise, unloading knee braces for patients with tibiofemoral unicondylar medial knee osteoarthritis can be used with the aim to reduce pain, joint stiffness, and medial compartment loading and enhance joint proprioception and functional stability [6,10,25,26]. These effects may also support the conduction of exercises, and in that case, unloader braces have the potential to serve as a treatment-supporting device. Despite limited evidence of the effectiveness of knee braces [6,8], the German guideline for the treatment of knee osteoarthritis recommends unloader knee braces as a “can do” option [7].

### Objectives

Considering that exercise is one of the core treatment options for knee osteoarthritis, as well as the fact that mHealth provides new opportunities to guide home-based exercise, and the potential benefit of unloader braces to support exercise conduction, this study aimed to investigate the efficacy of a 12-week mHealth app–supported exercise intervention (re.flex) with (intervention group [IG] AB) and without (IG A) a corrective knee brace in comparison to a control group (CG) on health-related outcomes in patients with moderate to severe unicondylar knee osteoarthritis. The primary outcome was the joint comparison of the 2 app-based study arms (IG A and IG AB) regarding osteoarthritis-specific pain (Knee Injury and Osteoarthritis Outcome Score [KOOS], pain subscale) versus the CG immediately after the 12-week intervention phase.

## Methods

### Study Design

This study was conducted as a randomized controlled superiority trial. Study participants were randomly assigned in a 1:1:2 ratio to an IG with 2 subgroup arms (app-based exercise training [IG A] and app-based exercise training in combination with a supportive knee brace [IG AB]) and a CG. The study is reported following the CONSORT-EHEALTH (Consolidated Standards of Reporting Trials of Electronic and Mobile Health Applications and Online Telehealth) checklist [27] and the Consensus on Exercise Reporting Template checklist for reporting exercise interventions [28].

### Ethical Considerations

Ethics approval was obtained from the ethics committee of the University Hospital Tübingen (550/2020BO). The participants signed a written informed consent and were given a study ID number. Identifiable information was stored on password-protected servers. There was no compensation for participation in the study. Study materials were provided to participants for free. The study was registered in the German Clinical Trials Register (DRKS00023269).

### Participants

Participants with knee osteoarthritis were recruited via advertisements in regional newspapers as well as emails sent to the employees of the University Hospital Tübingen and the University of Tübingen. Interested persons were screened for eligibility via phone call. Final inclusion or exclusion took place at the University Hospital Tübingen in the context of the medical examination at baseline. Inclusion and exclusion criteria are described in Textbox 1.

**Inclusion and exclusion criteria for study participants.**

**Inclusion criteria**

- Age of ≥18 years
- Knee osteoarthritis (self-reported according to the wording of the study questionnaire Gesundheit in Deutschland aktuell [29] and verified by the study physician at t0)
- Unicondylar tibiofemoral concerns
- Moderate to severe knee osteoarthritis assessed using the Knee Injury and Osteoarthritis Outcome Score, pain subscale (≤60 points at the time of screening)
- Knee osteoarthritis as the primary location of symptoms
- Access to a tablet or mobile phone with iOS operating system
- Willingness to use the app to exercise
- Willingness to wear a brace while exercising
- Informed consent for study participation

**Exclusion criteria**

- Scheduled or implanted knee joint replacement
- Osteoarthritis primarily located in the hip joint or a joint other than the knee
- Diffuse knee pain or retro-patellar pain only
- Concerns affecting physical performance in everyday life (measured using the Physical Activity Readiness Questionnaire [30,31] and verified by the study physician at t0)
- Concerns located at the back or lower extremities currently treated by a physician or health professional and other previous surgeries, injuries, or concerns that may impair measures of strength and balance or the exercise intervention itself
- Insufficient German language skills for self-administered questionnaires and app instructions during training

### Development of the Intervention

The re.flex intervention (Kineto Tech Rehab SRL) served as the underlying software and hardware for the intervention, including the basic app structure and the biofeedback via an avatar of the moving body part that is regulated by 2 accelerometers to guide and control training exercises. On the basis of this, a 12-week exercise program specifically designed for patients with knee osteoarthritis was developed by a team of experts of the Department of Sports Medicine at the University Hospital Tübingen. This program was then implemented into the app by the software manufacturer. Exercises were selected based on current knee osteoarthritis–specific recommendations from international guidelines [5,6,32] and years of experience by the study team in planning and conducting exercise interventions for patients with hip and knee osteoarthritis [33-38]. To test, analyze, and improve the re.flex knee osteoarthritis intervention during the development process, an iterative design approach [39] was used. In 2 test phases of 2 and 4 weeks, parts of the exercise program, as well as the app handling and usability, were tested by volunteers with knee concerns (data not published). Volunteers involved in the test phases were not included in the randomized controlled trial. In this study, the iOS app version 1.1.38 at the time of intervention completion of the IG was evaluated. During the intervention phase, minor technical bugs were fixed.

### App-Guided Exercise Intervention

The exercise intervention was a 12-week app-guided home training program specifically designed for patients with knee osteoarthritis.

#### App Features

**Figure 1:**
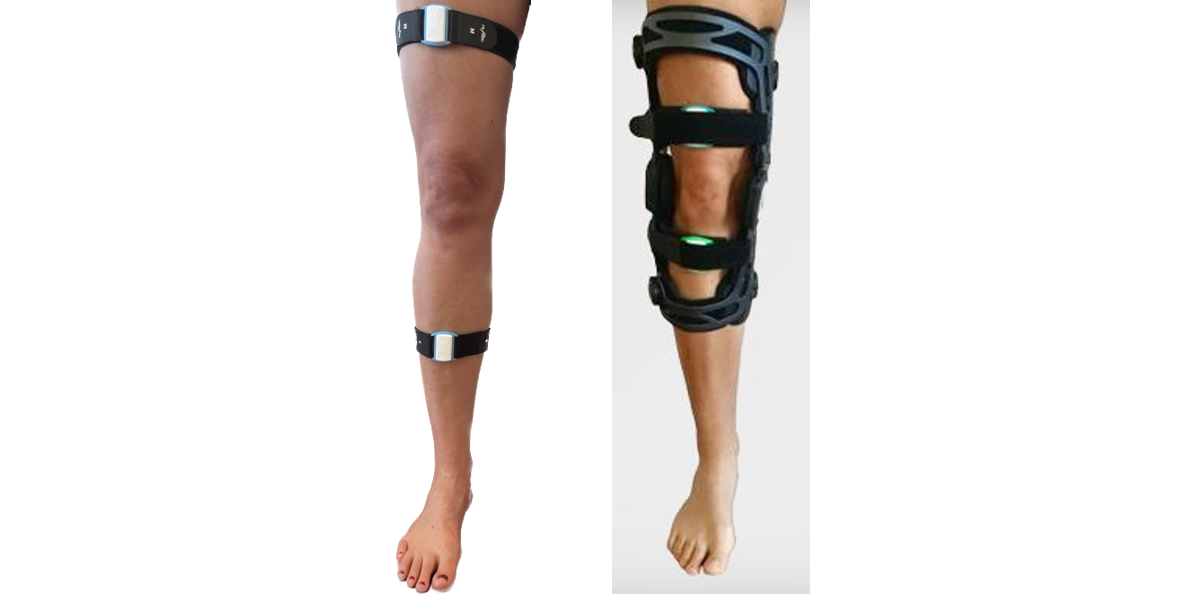
Re.flex technology directly attached to the lower limb (left) and Sporlastic GmbH GENUDYN OA SMART with re.flex technology (right).

**Figure 2:**
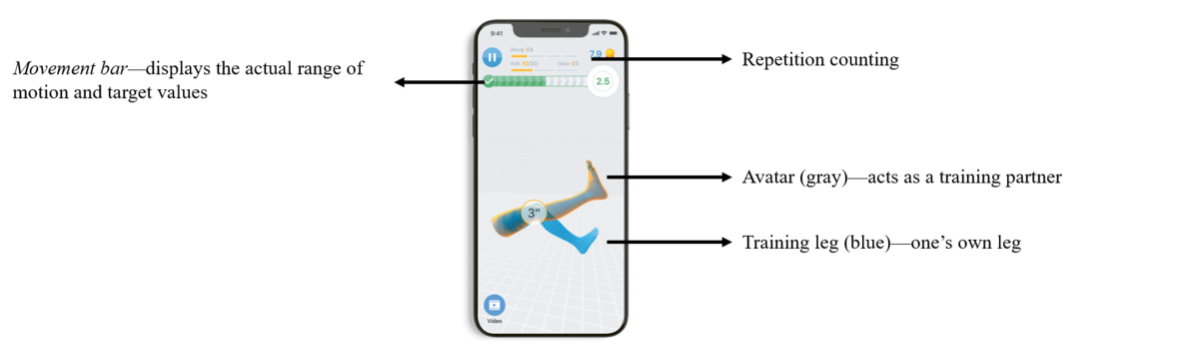
Screenshot with features of the re.flex app.

The re.flex app can be classified as a fully automated, digital health app including a training app and 2 accelerometers to monitor joint movement. It was used to guide and monitor the 12-week exercise intervention of this study. Sensors were attached proximally and distally to the affected knee joint or to the more affected joint (ie, signal joint) in case of bilateral knee osteoarthritis*.* They were directly attached to the skin using a hook-and-loop tape (IG A; Figure 1, left) or integrated into the brace (IG AB; Figure 1, right). Before each training session, sensors had to be calibrated by performing a movement task. The app acted as a virtual training partner, providing exercise descriptions and videos as well as setting the number of repetitions and sets of the exercises. Movement execution was monitored by the sensors and visualized via a blue avatar leg in the app interface. The blue avatar had to be aligned with another displayed gray avatar leg that moved according to the recommended movement velocity. A movement bar further visualized the current range of motion of the training leg. This bar served as an orientation on how far the leg should be moved in each direction relative to the starting position. If an exercise was not performed correctly, verbal instructions were given (eg, “extend your knee more”). After each set of exercises with the sensor-equipped leg, patients were called to conduct the set with the other leg as well. However, sets and repetitions of the other leg were performed autonomously and were not monitored in the log files of the app. Another feature of the app was to remind users of upcoming training sessions via push notifications. Figure 2 and the app manual (Multimedia Appendix 1) illustrate the structures and features of the re.flex app. The use of the app and sensors for IG A and IG AB was introduced after randomization at baseline by the pretrained study staff. Patients further received a user manual for software and hardware and log-in data for their personal anonymous and free app user account. The login data did not contain any personal data of the participants but used a fake email address with the patient’s pseudonym and an individual password. Only the authors of this study were able to reidentify them with their personal data.

#### Exercise Program

The progressively designed 12-week program included 3 sessions per week with 5 different exercises and a duration of 25 to 30 minutes each. The exercise poses differed between supine, sitting, and standing. The primary focus of the intervention was to strengthen knee extensors, knee flexors, and hip abductors. Furthermore, exercises aimed for mobilization, muscle stretching, and balance training. The required training material included a chair, a ball or a pillow, and the provided training bands with different resistance levels.

**Figure 3:**
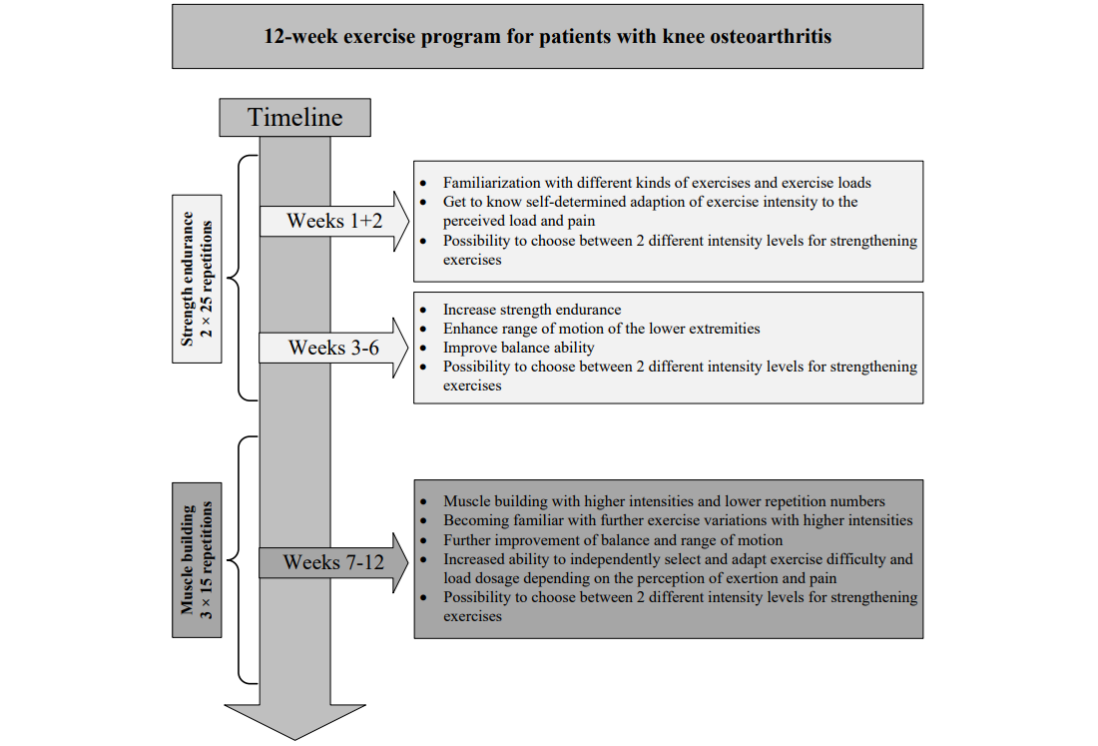
Objectives of the 12-week exercise program.

The first 2 weeks focused on familiarization with different kinds of exercises and exercise loads. In this regard, patients were able to adapt exercise intensity self-determinately according to perceived strain and pain, which were assessed after each set of exercises as well as before and after each training session using in-app scales. After the period of familiarization, the exercise sessions of the following 4 weeks were designed to increase strength endurance, enhance the range of motion of the lower extremities, and improve balance ability. From week 7 on, the intervention mainly focused on muscle building. Concurrently, the complexity of the balance tasks was increased accordingly by reducing the sensory input (eg, eyes closed) or modifying the supporting surface (eg, tandem stance). The exercises provided in 2 intensity levels were predefined for each session. An overview of the different phases and objectives within the 12-week exercise program is given in Figure 3. Throughout the intervention phase, users could contact the provider for technical and medical issues using the app messenger service. In the context of the study, this function was supervised by the study personnel.

#### Individual Exercise Dosing

At baseline, participants were instructed by the study staff to perform the last 2 to 3 repetitions of each set within a strenuous to very strenuous exertion level. During balance tasks, participants were instructed to perform the task properly at all times while still maintaining the challenge. To ease the fitting of the optimal intensity level, patients could always choose between 2 different intensity levels via an in-app button feature and could further vary the resistance of elastic exercise bands, if applicable. In addition to the intensity specifications, exercises should be performed in a pain-free to low-level pain range. The following instructions were given to the patients if they experienced increased pain during exercising: (1) check exercise performance and correct if necessary, (2) reduce training intensity by selecting an easier exercise variation or reducing the number of repetitions or sets, or (3) skip the exercise. Exercise adaptations in case of increased pain were prioritized versus intensity specifications. The information and guidance for training were provided both orally and written on a fact sheet.

### Knee Brace Intervention (Additional to Exercise)

The 2 exercise groups (IG A and IG AB) only differed with respect to the additional use of a corrective knee brace (GENUDYN OA SMART; Sporlastic GmbH) in IG AB. The brace works according to the 3-point principle and exerts pressure onto the unaffected condyle to correct the leg axis. Thus, it is indicated for patients with unicondylar concerns only. The use of the brace while exercising was mandatory. However, patients were free to use the brace in everyday life as well. Participants were asked to document the wear time of the brace in a paper-and-pencil study diary. The brace was worn at the knee joint affected by osteoarthritis or at the signal joint in case of bilateral knee osteoarthritis. It was fitted by an orthopedic technician during the baseline examination.

### CG Arm

Participants on the waiting list did not receive any study intervention or instruction for any change to their normal habits—“Just keep on like before.” They were allowed to make use of usual care provided by the treating physician, if applicable. Usual care was defined as any kind of prescribed pharmacological or physical interventions a patient with knee osteoarthritis usually receives when consulting a medical doctor because of knee osteoarthritis. These may include physical therapies such as regular physiotherapy, manual therapy, electrotherapy as well as orthotic devices, and medical prescriptions for pharmacological agents such as nonsteroidal anti-inflammatory drugs (NSAIDs). These reflect the relevant treatment options according to the current national guidelines in Germany [7]. Moreover, participants in this group were informed about the opportunity to make use of the app after the follow-up assessment.

### Outcomes

Data collection was conducted at baseline (t0) and at the 3-month follow-up (t3). Medical examinations and the outcome assessments of performance measures before and after the intervention (t3) took place on-site at the University Hospital Tübingen. Patient-reported outcome measures (PROMs) were assessed using web-based questionnaires (Questback GmbH). Questionnaires were activated on the days of assessment (t0 and t3), and study participants were asked to answer promptly. In case of delayed response, participants received a reminder via email.

### Patient Characteristics

Age, gender, BMI, medical history (eg, relevant diagnoses and previous injuries or surgeries at the lower extremities or lower back), previous experiences with strengthening exercises or hip or knee exercise groups, and technical affinity were determined at baseline (t0).

### Primary Outcome

The primary outcome measure was the joint comparison of the 2 app-based study arms (IG A and IG AB) versus the CG with regard to osteoarthritis-specific pain immediately after the 12-week intervention phase (t3). Pain was determined using the 10-item pain subscale of the KOOS [40,41]. The KOOS is a patient-reported outcome measurement instrument developed to assess the patient’s opinion on their knee and associated problems and uses a 5-point Likert scale. It evaluates short-term and long-term consequences of knee injuries and primary osteoarthritis in 5 separately scored subscales. Each subscale is transformed to a scale of 0 to 100 points, with a higher score reflecting a better health status.

### Secondary Outcomes

#### Overview of PROMs

Osteoarthritis-specific symptoms, physical function (activities of daily living), sport and recreation, and knee-related QoL were assessed using the other KOOS subscales. Patient-reported HRQoL was evaluated using the Veterans RAND 12-item Health Survey [42,43]. The Mental Component Score (MCS) and Physical Component Score (PCS) were calculated and used for further analysis. They both can adopt values in the range of 0 to 100 points. Higher scores indicate a better overall HRQoL. Patients’ fear of movement was determined using the 11-item German version of the Tampa Scale of Kinesiophobia [44], with a scoring range of 6 to 24 whereby a higher score indicates a greater fear of movement. Physical and sports activity of a typical week, including frequency and time spent on transportation-related cycling and sports, fitness, or recreational activities, were quantified using the European Health Interview Survey–Physical Activity Questionnaire [45]. Exercise-specific self-efficacy was examined using the 9-item multidimensional Self-Efficacy for Exercise Scale [46], which ranges from 0 (not safe at all) to 10 (absolutely safe). The scale was used as a total score and then further divided into 3 subscales: task, coping, and scheduling. Higher scores indicate a higher exercise-specific self-efficacy. Control competence for physical exercising is a subcompetency of the physical activity–related health competence model. It relates to the perceived competence to individually structure and control physical activity in a health-effective way. It is mainly based on action-related knowledge but also requires the ability to sense and interpret body signals (eg, to adjust intensities based on muscle soreness) [47]. Control competence for physical exercising was quantified using 6 items according to Sudeck and Pfeifer [47] and 4 self-constructed items specifically focusing on exercises for the lower limbs [15]. Each item was scored on a 4-point Likert scale ranging from 1 (totally disagree) to 4 (totally agree). The mean value of all items was used for analysis, with higher scores reflecting a higher level of control competence.

#### Performance Measures

Performance measures included isometric maximum strength measurement of the knee extensors and knee flexors using DAVID strength machines (F200 Leg Extension and F300 Leg Curl; Schupp GmbH & Co. KG). Knee extensor strength was measured at 60° knee flexion, and knee flexion strength was measured at 30° knee flexion. Before testing, participants were instructed to conduct 5 to 8 dynamic concentric repetitions of the target movement at 50% to 60% of maximum force and 2 isometric repetitions at submaximal force in the given test position. Participants were instructed not to provoke an increase in pain level during testing. All measures were taken twice for each leg, and the highest value was used for analysis. Relative values (Newton meters per kilogram of body weight) were reported.

The 30-second chair stand test [48] is an instrument to measure leg strength endurance. Participants were seated with a straight back in the middle of a chair (seat height: 17 inches; participants with a knee angle of <90° received a pad to increase chair height) with hands and arms crossed in front of the upper body. The feet were completely positioned on the floor. Participants were asked to stand up to full knee extension and then sit back again as many times as possible within 30 seconds. The total number of times the patient did come to a full standing position within the 30 seconds were counted. One complete movement execution was allowed before measurement.

Postural control tests were performed using a plantar pressure mat (zebris GmbH) to evaluate the course of the center of pressure in four different conditions: (1) bipedaled parallel stance with eyes open and (2) eyes closed, (3) bipedaled tandem stance with eyes open and the leg with knee osteoarthritis or signal joint in front and behind, and (4) one-legged stance with eyes open standing on the leg with knee osteoarthritis or signal joint. All tests were conducted in an upright position looking forward with both hands fixed at the superior iliac crest. After one test trial to become familiar with the procedure, conditions 1 and 2 were taken once, and conditions 3 and 4 were performed twice. The lowest value of each condition was used for analysis. The test duration was 10 seconds for conditions 1, 2, and 3 and 6 seconds for condition 4.

#### Adherence

Sensor- and app-based log files were read out for each exercise session separately and were used to quantify exercise adherence. Intervention finishers were defined as individuals who participated with >50% of overall exercise session adherence and were still active at weeks 11 and 12 of the intervention phase. Overall exercise session adherence was quantified by calculating the percentage of conducted exercise sessions relative to the overall number of prescribed exercise sessions irrespective of the adherence to the prescribed exercise dosage (number of sets and repetitions). Exercise repetition adherence was determined using the number of valid repetitions of all exercises of a session related to the prescribed repetitions. Percentage data were averaged across all exercise sessions (mean and SD). The active training time of an exercise session was calculated by adding all intervals between the time stamps of successive repetitions of an exercise unless the differences were of >60 seconds. If so, these data were not considered to exclude resting times within the active training time. The active training time was averaged across all exercise sessions (mean and SD) and further differentiated for weeks 1 to 6 and 7 to 12. The daily training time was the gross training time. It was calculated as the difference between the first and last repetition on a training day including all breaks, recalibrations, reviewing the exercise instructions, and the training of the other leg. For analysis of the active training time and gross training time, only cases with an exercise repetition adherence of 100% were considered. In addition, cases were excluded for which the gross training time was of >180 minutes as these cases indicate long breaks during the training or split training sessions and, thus, also possibly falsify pain and intensity data. To monitor perceived exercise intensity, participants were asked to rate their perceived overall exertion at the end of each exercise session. Perceived exertion was measured using the rate of perceived exertion scale from 0 (no exertion at all) to 10 (maximum conceivable exertion). Perceived pain before and after each exercise session was measured using the Faces Pain Scale [49]. Instead of numbers, 6 faces with different facial expressions were used to comment on perceived pain. Each face was associated with a textual reference for the pain intensity, and the faces were scored as 0 (no pain), 2 (little pain), 4 (moderate pain), 6 (much pain), 8 (very much pain), and 10 (highest imaginable pain). In the app, the 6 faces of the original version were replaced by standardized emojis of the iOS platform. For perceived exertion and pain analysis, only cases in which the training session was at least started (exercise repetition adherence of >0) were considered.

#### Concomitant Care

Concomitant pharmacological care (NSAIDs and analgesics) during the study phase was assessed retrospectively at t3.

#### Safety

Participants included in the study were asked to document all adverse events (AEs) that occurred during the study period. Participants in the IGs were further instructed to interrupt the training program in case of any suspicious symptoms, fatigue, or severe pain during exercising or wearing the knee brace. Mild AEs had to be reported to the responsible study staff within 1 week (via email, phone, or in-app support chat). AEs that required referral to a physician or other health care professional had to be reported immediately. The decision on how to proceed was up to the study physician as well as the study director (sports scientist and physiotherapist) and referred to the options of complete or temporary discontinuation or modification of the training regime. During data analysis, the reports were classified into AEs and serious AEs (SAEs; events related to death, life-threatening illness or injury, or inpatient hospitalization). They were further classified into expected and unexpected events, and the link to the intervention was differentiated into *sure*, *likely*, *possible*, *unlikely*, or *none*. Actions taken were classified into need or no need for immediate medical care (eg, referral to orthopedist, physiotherapy, or medication) and change in intervention modalities (eg, modification, pausing, stopping, or none).

### Sample Size

Sample size was calculated based on an a priori power analysis (PASS 2020; NCSS, LLC). The sample size estimation was related to the primary outcome regarding the comparison of the 2 app-guided IGs (independently of the possible supplementary use of a knee brace in IG AB) versus the CG. Furthermore, the sample size estimation was based on the following assumptions: α level of.05, power of β=.8, and a correlation of pretest-posttest values of *r*=0.5 [50]. Standardized effect sizes (ESs) were used due to a lack of studies with comparable interventions and measures of dispersion. In this regard, required sample sizes to prove ESs between *f*=0.2 (equal to Cohen *d*=0.4) and *f*=0.4 (equal to Cohen *d*=0.8) were calculated with n=14 for *f*=0.2 and n=51 for *f*=0.4. Under the aforementioned assumptions and an expected dropout rate of approximately 15%, 30 participants should be recruited into each group (IG and CG) to verify a medium ES of *f*=0.2.

### Randomization

Before study start, a randomization list was created using computer-generated random numbers (0 and 1) in 7 blocks with 10 slots each. Subsequently, sealed envelopes were prepared containing sequential numbers corresponding to the group assignment resulting from the randomization list. Randomization into the IG and CG in a 1:1 ratio took place after baseline testing (t0) in order of appointment (eg, the first patient received the envelope with the first lot and so on). Participants allocated to the IG were then again randomized in a 1:1 ratio to 1 of the 2 intervention subgroups, IG A or IG AB, using the aforementioned procedure, including the prestudy preparation of the randomization list and sealed envelopes as outlined previously. The sealed envelopes were handed over by the study personnel. The randomization list and the sealed envelopes were prepared by a person not involved in the conduction, assessment, or data analysis of the study. Participants were not randomized in case of exclusion before completion of the baseline examination at t0.

### Blinding

Participants and study personnel responsible for data collection and data analysis were not blinded to the group assignment or type of study intervention.

### Statistical Analysis

Baseline characteristics of the IG (IG A and IG AB) and CG study groups at t0 were described using descriptive statistics, with continuous data being presented as mean and SD or median and IQR and categorical variables being presented as absolute numbers and percentages. Unpaired Student 2-tailed *t* tests or Mann-Whitney *U* tests (in case the normal distribution of the data were violated) and Pearson chi-square tests for categorical data were used to compare baseline characteristics in the IG and CG. The primary outcome was evaluated using an analysis of covariance (ANCOVA) with the dependent variable at t3 (KOOS pain subscale at t3), the fixed-effect group (IG and CG), and the covariate (KOOS pain subscale at t0) to adjust for baseline values. Secondary outcomes for the comparison of the IG and CG were handled accordingly. Mann-Whitney *U* tests using the difference t3–t0 of the variable of interest were calculated as a nonparametric alternative. Imputation of missing data was not foreseen. The α level was set to .05. Adjustments for multiple testing were applied for the tandem stance to account for 2 test conditions using Bonferroni correction (α=.025). Data were analyzed as randomized (intention to treat) following the complete case analysis approach. Sensitivity analyses regarding the evaluations of the 5 KOOS subscales (primary analysis and additional explorative analysis) using the last observation carried forward method and the mean imputation method were conducted to replace missing data and, thus, control for any possible bias due to missing values. In case of no significant deviation compared to the complete case analyses, the analyses were continued as initially intended. Between-group ESs were calculated according to Olejnik and Algina [51], with the differences of the adjusted postintervention values divided by the pooled SD at t3 (original data) and interpreted according to Cohen. Thereby, ESs of 0.2 to <0.5 were interpreted as small, ESs of 0.5 to <0.8 were interpreted as medium, and ESs of ≥0.8 were interpreted as large. Data were analyzed using the software packages Microsoft Excel (Microsoft Corp) and SPSS Statistics (IBM Corp).

### Subgroup Analysis

In addition to the aforementioned pooled comparison of the IG versus the CG, explorative separate subgroup comparisons of IG A and IG AB versus the CG as well as comparisons of IG A versus IG AB were conducted for the KOOS at t3. ANCOVA was used according to the procedure described previously.

## Results

### Participant Flow

**Figure 4:**
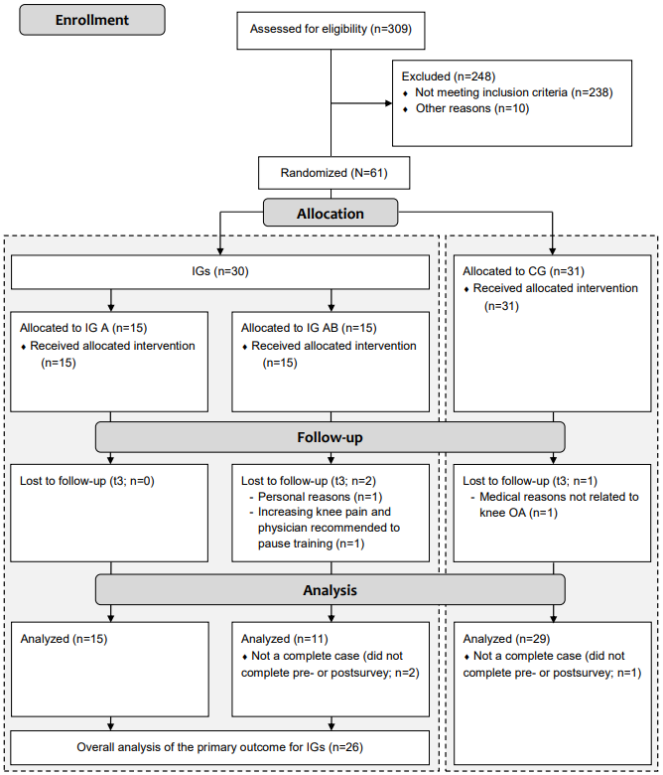
Participant flowchart. CG: control group; IG: intervention group; IG A: app-based training; IG AB: app-based training+brace; OA: osteoarthritis.

Details on participant flow are outlined in Figure 4. Recruitment started on September 21, 2020. All patients were enrolled within 2 weeks, with the first patient included on October 12, 2020. The follow-up after the 12-week intervention period was completed on January 27, 2021. In total, 61 participants were randomized. Thereof, of the 61 participants, 30 (49%) were allocated to the IG (re.flex; n=15, 50% into IG A and IG AB each), and 31 (51%) were assigned to the CG (usual care). Loss to follow-up was 7% (2/30) for the IG and 3% (1/31) for the CG. In addition, 3 complete data sets were excluded from analysis (listwise case exclusion) due to surveys not completed at t0 or t3. Finally, 87% (26/30) of the participants from the IGs and 94% (29/31) of the participants from the CG were considered in the analysis of the primary outcome.

### Baseline Data

Table 1 reports the sociodemographic and outcome-related baseline values of the participants. At t0, none of the variables showed a statistically significant difference between the IG and CG. Overall, the gender distribution of the participants was balanced (31/61, 51% male and 30/61, 49% female), mean age was 62.9 (SD 8.5) years, and mean BMI was 27.7 (SD 4.5) kg/m^2^. However, it has to be noted that the number of male participants was higher in the IG and, on average, the participants in this group were also younger. Most of the participants (58/61, 95%) had never taken part in a hip or knee exercise group before. IG A and IG AB showed statistically significant differences at baseline for the one-legged stance of the signal joint (*P*=.02), with higher mean values for IG A.

**Table 1:** Baseline data for the complete case sample in total as well as differentiated according to group assignment (N=61).

Characteristic		Total (N=61)	IG^a^ (n=30)	CG^b^ (n=31)	*P* value^c^	
**Gender, n (%)**						.16
	Men	31 (51)	18 (60)	13 (42)		
	Women	30 (49)	12 (40)	18 (58)		
Age (y), mean (SD)		62.9 (8.5)	61.5 (7.5)	64.2 (9.3)	.21	
BMI (kg/m^2^), mean (SD)		27.7 (4.5)	27.5 (5.0)	27.9 (4.2)	.75	
**Education, n (%)^d^**						.34
	Academic education	32 (53)	13 (45)	19 (61)		
	Vocational education	27 (45)	15 (52)	12 (39)		
	No vocational education	1 (2)	1 (3)	0 (0)		
**Employment, n (%)^d^**						.44
	Employed	30 (50)	16 (55)	14 (45)		
	Retired	30 (50)	13 (45)	17 (55)		
**Previous experience with exercise therapy, n (%)**						.51
	Very high	5 (8)	2 (7)	3 (10)		
	High	13 (21)	8 (27)	5 (16)		
	Moderate	30 (49)	15 (50)	15 (48)		
	Low	11 (18)	3 (10)	8 (26)		
	Very low	2 (3)	2 (7)	0 (0)		
**Previous participation in a hip or knee sports group, n (%)**						.54
	Yes	3 (5)	2 (7)	1 (3)		
	No	58 (95)	28 (93)	30 (97)		
Technical affinity^e^, mean (SD)		2.6 (0.6)	2.5 (0.7)	2.7 (0.6)	.25	
**KOOS^f^, mean (SD)**						
	Pain	53.7 (15.9)	51.0 (15.8)	56.2 (15.8)	.22	
	Symptoms	56.7 (17.4)	54.3 (17.7)	58.9 (17.1)	.33	
	Physical function (ADLs^g^)	70.4 (17.2)	68.9 (15.6)	71.8 (18.8)	.54	
	Sport and recreation	33.7 (21.2)	33.9 (21.5)	33.6 (21.3)	.97	
	QoL^h^	39.0 (15.2)	38.7 (15.7)	39.2 (15.0)	.90	
**Health-related QoL^i^, mean (SD)**						
	PCS^j^	38.2 (9.3)	37.3 (9.0)	39.0 (9.7)	.51	
	MCS^k^	55.2 (9.3)	54.6 (10.9)	55.7 (7.9)	.67	
**Exercise-specific self-efficacy^l^**						
	Overall, median (IQR)^m^	8.4 (1.9)	8.7 (2.3)	8.3 (1.5)	.79	
	Task efficacy, median (IQR)^m^	8.3 (2.7)	8.5 (2.8)	8.3 (2.3)	.60	
	Coping efficacy, mean (SD)	7.7 (1.8)	7.7 (2.2)	7.7 (1.5)	.97	
	Scheduling efficacy, median (IQR)^m^	9.7 (1.7)	9.5 (2.1)	9.7 (1.2)	.20	
Control competence^n^, mean (SD)		3.0 (0.7)	3.1 (0.7)	3.0 (0.6)	.50	
Fear of movement^o^, mean (SD)		10.7 (3.7)	10.9 (3.9)	10.4 (3.5)	.62	
Aerobic physical activity^m,p^ (minutes per week), median (IQR)		300.0 (425.0)	275.0 (435.0)	345.0 (402.5)	.56	
**Isometric maximum force measurement^q^**						
	Knee extension (N m/kg), mean (SD)	1.2 (0.5)	1.3 (0.5)	1.1 (0.4)	.08	
	Knee flexion (N m/kg), median (IQR)^m^	1.02 (0.5)	1.1 (0.5)	1.0 (0.3)	.13	
30-second chair stand (repetitions)^m,r^, median (IQR)		10.0 (3.0)	10.0 (4.0)	10.0 (3.0)	.18	
**Postural control—COP^s^ path (mm)**						
	Bipedaled parallel stance (eyes open), median (IQR)^m,t^	47.1 (29.1)	45.5 (31.4)	49.8 (39.5)	.10	
	Bipedaled parallel stance (eyes closed), median (IQR)^m,u^	86.3 (53.4)	88.5 (50.5)	83.5 (72.5)	.41	
	Bipedaled tandem stance with signal joint leg in front, mean (SD)^v^	245.6 (91.4)	235.7 (98.0)	256.2 (84.2)	.42	
	Bipedaled tandem stance with signal joint leg at the back, median (IQR)^m,w^	222.1 (120.1)	213.2 (110.4)	224.1 (128.4)	.64	
	One-legged stance of signal joint, mean (SD)^x^	184.4 (75.2)	182.5 (78.7)	186.4 (72.8)	.86	

^a^IG: intervention group.

^b^CG: control group.

^c^The *P* value related to the comparison of the IG versus the CG.

^d^n=1 missing value.

^e^5-point Likert scale from 1 (*not true at all*) to 5 (*fully true*); n=6 missing values.

^f^KOOS: Knee Injury and Osteoarthritis Outcome Score. Scored from 0 to 100, with higher scores reflecting a better health status; n=6 missing values.

^g^ADL: activity of daily living.

^h^QoL: knee-related quality of life.

^i^Scored from 0 to 100, with higher scores reflecting a better health-related QoL; n=6 missing values.

^j^PCS: Physical Component Score.

^k^MCS: Mental Component Score.

^l^10-point scale from 0 (*not safe at all*) to 10 (*absolutely safe*); n=6 missing values.

^m^In case of nonparametric testing, median and IQR were reported.

^n^4-point Likert scale from 1 (*totally disagree*) to 4 (*totally agree*); n=6 missing values.

^o^Scored from 6 (*no fear*) to 24 (*extreme fear*); n=6 missing values.

^p^n=6 missing values.

^q^n=4 missing values.

^r^Number of counted repetitions; n=4 missing values.

^s^COP: center of pressure.

^t^n=5 missing values.

^u^n=4 missing values.

^v^n=7 missing values.

^w^n=8 missing values.

^x^n=11 missing values.

### Primary Outcome

**Figure 5:**
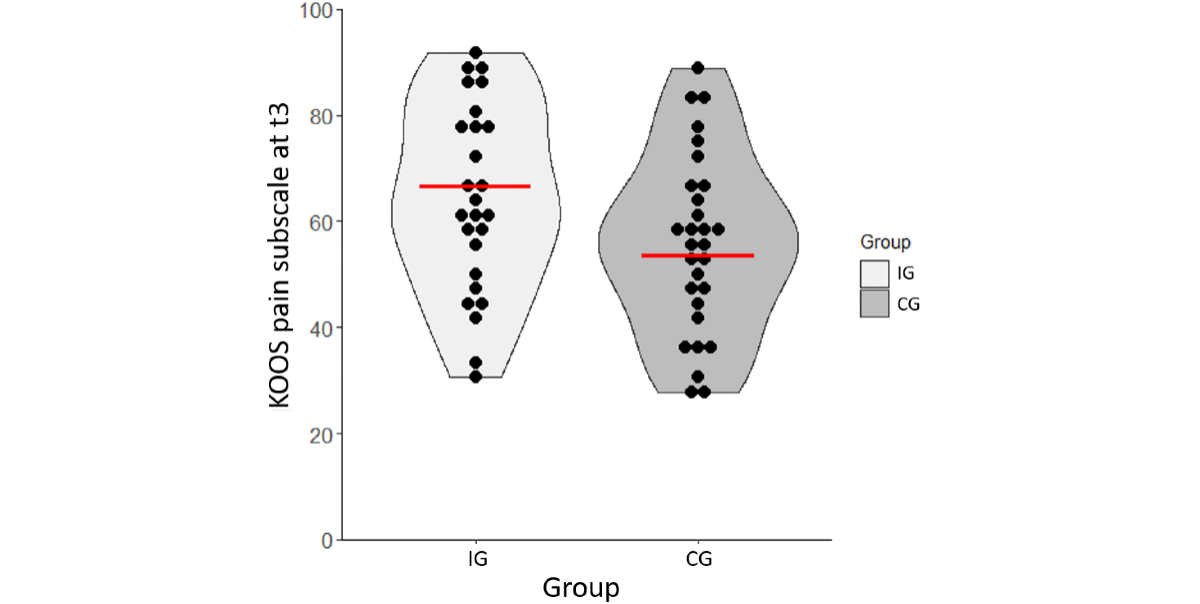
Knee Injury and Osteoarthritis Outcome Score (KOOS) pain subscale at 3 months (t3) of the intervention group (IG) and control group (CG). Violin plots display data distribution (tails are trimmed to the range of the data) and baseline-adjusted means (red line).

Table 2 and Figure 5 present the primary outcome, the KOOS pain subscale, at 3 months. ANCOVA showed a statistically significant between-group effect (*F*_1, 52_=20.01; *P*<.001; ƞ^2^=0.278), with greater pain reduction for the IG compared to the CG. The baseline-adjusted mean difference was 13.2 points, demonstrating a medium effect in favor of the IG (ES=0.76).

**Table 2:** Primary and secondary outcome measures.

Outcome measure and group				Mean (SEM)		Mean difference (IG^a^–CG^b^; 95% CI)^c^	*P* value	ES^d^
				t0^e^	t3^f^			
**Patient-reported outcome measures**								
	**KOOS^g^ (score of 0-100; worst to best)**							
		**Pain subscale**				13.2 (7.3 to 19.1)	<.001	0.76
			IG (n=26)	51.0 (3.1)	66.7 (2.1)			
			CG (n=29)	56.2 (2.9)	53.5 (2.0)			
		**Symptoms subscale**				10.0 (2.4 to 17.5)	.01	0.53
			IG (n=26)	54.3 (3.5)	65.1 (2.7)			
			CG (n=29)	58.9 (3.2)	55.2 (2.6)			
		**Physical function (ADLs^h^) subscale**				12.0 (5.9 to 18.1)	<.001	0.64
			IG (n=26)	68.9 (3.1)	79.5 (2.2)			
			CG (n=29)	71.8 (3.5)	67.5 (2.1)			
		**Sport and recreation subscale**				10.7 (1.9 to 19.5)	.02	0.47
			IG (n=26)	33.8 (4.2)	48.2 (3.2)			
			CG (n=29)	33.6 (4.0)	37.5 (3.0)			
		**QoL^i^ subscale**				12.5 (6.8 to 18.1)	<.001	0.76
			IG (n=26)	38.7 (3.1)	47.6 (2.0)			
			CG (n=29)	39.2 (2.8)	35.1 (1.9)			
	**Health-related QoL (score of 0-100; worst to best)**							
		**PCS^j^**				6.0 (2.8 to 9.2)	<.001	0.74
			IG (n=26)	37.3 (1.8)	44.0 (1.2)			
			CG (n=29)	39.0 (1.8)	38.0 (1.1)			
		**MCS^k^**				−2.6 (−6.2 to 1.0)	.15	—^l^
			IG (n=26)	54.6 (2.1)	53.0 (1.3)			
			CG (n=29)	55.7 (1.5)	55.6 (1.2)			
	**Exercise-specific self-efficacy (score of 0-10)**							
		**Overall**				0.1^m^ (−0.8 to 1.0)	.44	—
			IG (n=26)	8.1 (0.4)	7.7 (0.4)^n^			
			CG (n=29)	8.2 (0.2)	7.6 (0.3)^n^			
		**Task efficacy**				0.8^m^ (−0.2 to 1.8)	.13	—
			IG (n=26)	8.1 (0.4)	8.1 (0.4)^n^			
			CG (n=29)	7.8 (0.3)	7.3 (0.3)^n^			
		**Coping efficacy**				0.4 (−0.3 to 1.2)	.24	—
			IG (n=26)	7.7 (0.4)	7.2 (0.3)			
			CG (n=29)	7.7 (0.3)	6.7 (0.3)			
		**Scheduling efficacy**				−0.9^m^ (−1.8 to 0.0)	.42	—
			IG (n=26)	8.6 (0.4)	7.8 (0.4)^n^			
			CG (n=29)	9.2 (0.2)	8.7 (0.2)^n^			
	**Control competence (score of 1-4)**					0.2 (−0.0 to 0.3)	.09	—
		IG (n=26)		3.1 (0.1)	3.1 (0.1)			
		CG (n=29)		3.0 (0.1)	2.9 (0.1)			
	**Fear of movement (score of 6-24)**					−1.6 (−3.3 to 0.1)	.06	—
		IG (n=26)		10.9 (0.8)	9.9 (0.6)			
		CG (n=29)		10.4 (0.7)	11.5 (0.6)			
	**Aerobic physical activity (min/wk)**					150.5^m^ (13.7 to 287.4)	.28	—
		IG (n=26)		451.2 (97.6)	388.3 (62.9)^n^			
		CG (n=29)		382.2 (66.2)	237.8 (31.6)^n^			
**Performance measures**								
	**Muscle strength**							
		**Isometric maximum force—knee extension (N m/kg)**				0.0 (−0.1 to 0.2)	.59	—
			IG (n=28)	1.3 (0.1)	1.3 (0.1)			
			CG (n=29)	1.1 (0.1)	1.3 (0.1)			
		**Isometric maximum force—knee flexion (N m/kg)**				0.2^m^ (−0.1 to 0.5)	.40	—
			IG (n=28)	1.2 (0.1)	1.2 (0.1)^n^			
			CG (n=29)	1.0 (0.1)	1.0 (0.1)^n^			
		**30-second chair stand test (repetitions)**				1.8^m^ (0.2 to 3.4)	.23	—
			IG (n=28)	10.6 (0.6)	12.3 (0.5)^n^			
			CG (n=29)	9.6 (0.5)	10.5 (0.6)^n^			
	**Postural control**							
		**Bipedaled parallel stance (eyes open; COP^o^ path in mm)**				2.2^m^ (−11.0 to 15.4)	.08	—
			IG (n=28)	43.3 (3.3)	57.3 (4.9)^n^			
			CG (n=28)	52.3 (4.3)	55.1 (4.4)^n^			
		**Bipedaled parallel stance (eyes closed; COP path in mm)**				−23.2^m^ (−53.4 to 7.0)	.22	—
			IG (n=28)	89.4 (8.0)	92.2 (9.8)^n^			
			CG (n=29)	101.9 (12.5)	115.4 (11.4)^n^			
		**Bipedaled tandem stance with signal joint in front (COP path in mm)**				11.0 (−30.6 to 52.6)	.60^p^	—
			IG (n=28)	235.7 (18.5)	263.4 (14.3)			
			CG (n=26)	256.2 (16.5)	252.4 (14.9)			
		**Bipedaled tandem stance with signal joint at the back (COP path in mm)**				49.8^m^ (−100.9 to 1.3)	.048^p^	—
			IG (n=27)	236.6 (18.1)	218.5 (14.1)^n^			
			CG (n=26)	248.4 (16.9)	268.3 (21.4)^n^			
		**One-legged stance of signal joint (COP path in mm)**				−6.5 (−30.1 to 17.1)	.58	—
			IG (n=26)	182.5 (15.4)	177.1 (8.1)			
			CG (n=24)	186.4 (14.9)	183.6 (8.5)			

^a^IG: intervention group.

^b^CG: control group.

^c^Reporting the baseline-adjusted means.

^d^ES: effect size; only calculated for significant results.

^e^t0: baseline.

^f^t3: 12 weeks after baseline.

^g^KOOS: Knee Injury and Osteoarthritis Outcome Score.

^h^ADL: activity of daily living.

^i^QoL: quality of life.

^j^PCS: Physical Component Score.

^k^MCS: Mental Component Score.

^l^Not applicable.

^m^Mann-Whitney *U* test comparing the within-group differences t3–t0.

^n^Reporting unadjusted means; prerequisites for analysis of covariance not given.

^o^COP: center of pressure.

^p^Adjusted for multiple testing (*P*<.03).

### Secondary Outcomes

#### PROMs and Performance Measures

Secondary outcome measures at 3 months are outlined in Table 2. At t3, statistically significant differences between the IG and the CG were observed for all additional KOOS subscales in favor of the IG (symptoms: *F*_1, 52_=7.01, *P*=.01, and η^2^=0.119; physical function [activities of daily living]: *F*_1, 52_=15.56, *P*<.001, and η^2^=0.230; sport and recreation: *F*_1, 52_=5.98, *P*=.02, and η^2^=0.103; QoL: *F*_1, 52_=19.87, *P*<.001, and η^2^=0.277), as well as for the HRQoL PCS (*F*_1, 52_=13.94; *P*<.001; ƞ^2^=0.211). Baseline-adjusted mean differences between the IG and CG were between 10.0 and 12.5 points for the KOOS subscales and 6.0 points for the PCS. Interpreted according to the Cohen *d*, the intervention showed a medium treatment effect for improvements in patients’ symptoms, physical function, knee-related QoL, and the physical component of the HRQoL and a small treatment effect for sport and recreation activities. No statistically significant differences between groups were observed for any other secondary patient-reported or performance-related outcome measures.

#### Subgroup Analyses

The exploratory subgroup analyses between IG A and IG AB demonstrated baseline-adjusted mean differences ranging from 4.7 to 12.1 points for the 5 KOOS subscales in favor of the IG AB group. However, between-group differences were not statistically significant. Subgroup analyses for IG A and IG AB separately versus the CG showed superiority of IG AB versus the CG in all KOOS subscales as well as superiority of the IG A versus the CG for pain, physical function, and QoL. Table S1 in Multimedia Appendix 2 provides more details.

#### Adherence

Of the 30 participants in the IG, 27 (90%) were defined as intervention finishers. During the intervention phase, one participant dropped out due to personal reasons, and another dropped out because of non–device-related increasing knee pain and a subsequent physician-recommended pausing of the training intervention. One participant ceased the intervention from week 8 onward for unknown reasons. Overall, exercise session adherence for intervention finishers was 92.5% (899/972), indicating the percentage of training sessions that were at least started. In total, 7.5% (73/972) of all training sessions were not performed (adherence=0%). The overall mean exercise repetition adherence was 86.8% (SD 28.9%). The average active training time and gross training time in weeks 1 to 12 (total) was 15.5 (SD 4.1) minutes and 31.7 (SD 17.1) minutes, respectively. On average, participants reported a perceived pain score of 0.9 (SD 1.4) points before and 1.1 (SD 1.5) points after the exercise sessions. This was according to the Faces Pain Scale, equivalent to a slight traction or mild pain. The mean difference from after to before exercise sessions was 0.2 points. After the exercise sessions, the participants reported an average intensity value of 3.5 (SD 1.4) points. According to the rate of perceived exertion scale, this indicates a slight to somewhat exhausting load. Further details on adherence, training time, exertion, and pain outcomes are outlined in Tables 3 and 4.

**Table 3:** Adherence and training time.

Outcome measure	Weeks 1-12	Weeks 1-6	Weeks 7-12
Overall exercise session adherence (%)^a^	92.5	93.0	92.0
Exercise repetition adherence (%), mean (SD)^a^	86.8 (28.9)	87.6 (28.0)	85.9 (29.8)
Active training time (min), mean (SD)	15.5 (4.1)	17.6 (3.3)^b^	13.3 (3.7)^c^
Gross training time (min), mean (SD)	31.7 (17.1)	36.2 (19.3)^b^	27.3 (13.2)^c^

^a^Data refer to 972 exercise sessions (12 weeks with 3 sessions per week; n=27 [n=3 no intervention finishers]).

^b^Data out of 360 exercise sessions.

^c^Data out of 364 exercise sessions.

**Table 4:** Perceived exertion and pain outcomes.

Outcome measure	Values, mean (SD)	Values, median (IQR)	Range
Perceived exercise intensity after exercise sessions, mean (SD)^a,b^	3.5 (1.4)	3.0 (1.0)	3.5-9.0
Perceived pain before exercise sessions, mean (SD)^b,c^	0.9 (1.4)	0.0 (2.0)	0.9-6.0
Perceived pain after exercise sessions, mean (SD)^b,c^	1.1 (1.5)	0.0 (2.0)	1.1-6.0

^a^10-point scale from 0 (*no exertion at all*) to 10 (*maximum conceivable exertion*).

^b^Data out of 888 exercise sessions.

^c^10-point scale from 0 (*no pain*) to 10 (*highest imaginable pain*).

#### Concomitant Care

Concomitant pharmacological care data during the study phase were available for 93% (28/30) of the IG participants and 97% (30/31) of the CG participants. Overall, at t3, a total of 14% (4/28) of the IG participants compared to 10% (3/30) of the CG participants reported a daily intake of NSAIDs or analgesics during the study phase, and 7% (2/28) of the IG participants compared to 7% (2/30) of the CG participants reported a weekly intake of NSAIDs or analgesics during the study phase.

#### Safety and Technical Issues

No SAEs were reported throughout the intervention phase to the study personnel. In summary, 7 AEs were reported. In total, 4 of the AEs were sure to be intervention related (AEs number 11, 21, 40, and 59), of which 3 (75%) required a modification of the training (AEs number 11, 40, and 59) and 1 (25%) required pausing the training intervention (AE number 21). Medical care was necessary in one case (AE number 40). The remaining 43% (3/7) of the AEs were not intervention related (AEs number 29, 44, and 49). Further details are outlined in Table 5.

**Table 5:** Adverse events (AEs) throughout the study period.

ID	Group	Harm	Type	Expectation	Link to intervention	MC^a^	CoI^b^
11	IG^c^	Increased pain due to device-initiated overload (repetition count failure and range of motion)	AE	UE^d^	Sure	No	Modification
21	IG	Increased pain and feeling of permanent muscle soreness in legs and arms	AE	EE^e^	Sure	No	Pausing
29	IG	Training interruption due to lumbago	AE	—^f^	No	No	Pausing
40	IG	Increased pain (especially for standing exercises on one leg) and activated OA^g^	AE	UE	Sure	Yes	Modification
44 (DO^h^)	CG^i^	Dizziness and personal health problems	AE	—	No	Yes	None
49 (DO)	IG	Fall on knee and subsequently irritated and overloaded knee	AE	—	No	Yes	Stopping
59	IG	Increased pain and knee joint unusually warm	AE	UE	Sure	No	Modification

**^a^**MC: medical care; need for immediate medical care (yes) or no need for medical care (no).

^b^CoI: change of intervention.

^c^IG: intervention group.

^d^UE: unexpected event.

^e^EE: expected event.

^f^Not applicable or no link.

^g^OA: osteoarthritis.

^h^DO: dropout.

^i^CG: control group.

Technical issues reported by participants when using the app were used for minor technical bug fixes during the study period as well as for adjustments after the study period. In the following paragraph, only the summarized incident report of a participant on a specific topic is considered. On the one hand, bug fixes related to failures of the movement sensors, which, in some cases, did not adequately recognize patients’ movements and, thus, led to incorrect counting of exercise repetitions. This was the case for the following exercises: wall slide (n=12), knee extension exercises (seated and supine position; n=7), and hip abduction exercises (seated and standing position; n=5). Other problems included the need for multiple recalibrations during an exercise session (n=17), an incorrect representation of the training leg on the app (twisted and no reaction; n=12), and initial problems connecting the sensors (n=7).

## Discussion

### Principal Findings

#### Overview

This pilot study aimed to evaluate the efficacy of a 12-week app-based exercise intervention with or without an additional knee brace on symptoms and function in participants with knee osteoarthritis. The results of the study demonstrated small to medium treatment effects with statistically significant reductions in self-reported osteoarthritis-related pain (primary outcome) and other osteoarthritis-specific concerns (KOOS symptoms, physical function, sports and recreation, and QoL subscales), as well as an increase in the PCS of the general HRQoL after the 12-week app training versus the CG. No intervention effects were found for any other of the secondary outcomes. The intervention showed an excellent adherence rate and no SAEs.

#### Overview of PROMs

Previous studies have shown superiority of exercise interventions guided by fully automated mobile apps versus control to reduce knee-related pain and improve physical function in patients with knee osteoarthritis [22,23,52]. However, in contrast to our results indicating medium ESs of >0.6 for pain and physical function, Bossen et al [52], who focused their intervention primarily on increasing general physical activity, demonstrated much smaller effects of 0.2 for pain and physical function. Regarding the absolute differences between the baseline-adjusted postmeasures of the IG and CG, substantially higher between-group differences (10.0 to 13.2 points) were reported in this study in comparison to other studies with digital interventions (2.9 to 7.7 points), of which only Mecklenburg et al [23] used a similar sensor-assisted and app-based exercise program [22,23,52]. Uesugi et al [53] did not find a significant between-group effect at all.

From a clinical perspective, within-group differences for pain in the IG of our study exceeded the minimal clinically important improvement (MCII) of 8.7 points/100 as reported for patients with knee osteoarthritis who underwent a 12-week rehabilitation intervention with active and passive therapeutic treatments. Reported cutoff values for physical function (13.4 points/100) could not be reached [54]. However, regarding minimal clinically important differences (MCIDs) between the IG and CG, the ESs of our study are within or above reported thresholds for MCIDs [55]. Nevertheless, there are many different calculation methods for determining MCII and MCID, and these values may also differ between population groups and interventions, leading to a lack of consensus on which cutoff values should be used. Therefore, future studies should apply an own anchor-based approach to be able to define intervention-related MCID and MCII values for pain and physical function.

At present, nondigital interactions are the gold standard for exercise guidance, and novel interventions should not only be compared between each other but also in reference to the standards. Studies on nondigital interactions have reported small to medium effects in terms of pain reduction and improvement in physical function for delivery modes such as one-on-one treatments, class-based programs, and home-based exercises [9,56]. Verhagen et al [57] recently stated that the estimated effects regarding pain reduction of supervised exercising are very robust and no further intervention studies in this domain would change these findings. This is in contrast to cutting-edge results of an individual patient data meta-analysis on exercise therapy in knee and hip osteoarthritis questioning the clinical importance of reported effects versus those of a CG [58]. Accordingly, it seems reasonable to expand the field of application and investigate alternative, innovative ways of delivering exercise therapy that may further increase our knowledge of the effectiveness and mechanisms of action of new treatment delivery opportunities. Considering this, the app-based instruction appears to be a promising evolution of the currently proven standard therapy, especially as the results of this study indicate clinically important ESs. Another possible reason for the larger effects reported in our study may be related to the fact that we only included patients with at least moderate knee osteoarthritis symptoms. This reduced the potential risk of ceiling effects as the possible range of improvements for patients with early or mild disease-specific symptoms was much lower and baseline pain has been described as a moderator for treatment effectiveness [58].

The exploratory subgroup analyses for the KOOS subscales showed superiority of IG AB versus the CG in all KOOS subscales as well as superiority of IG A versus the CG for pain, physical function, and QoL. The direct comparison of both IGs provided a first indication of the superiority of IG AB versus IG A. However, these findings were not statistically significant. This potential trend of superiority of IG AB must be considered with caution due to the small subgroup sample size and should be verified in a subsequent data analysis with a larger sample.

Benefits of exercise therapy on mental and physical HRQoL in patients with knee osteoarthritis have also been reported in a recent meta-analysis with reported standardized mean differences of 0.52 for the PCS and 0.44 for the MCS [59]. Compared with these values, this study showed even greater improvements for the PCS with an ES of 0.74, yet no improvements in the MCS were observed. However, baseline scores for the MCS already exceeded the US population norm of 50 points [60].

When looking at health-psychological measures, participants in the IG had less fear of movement after the intervention in comparison to participants in the CG, although this finding failed to reach statistical significance. Mean values at baseline already indicated a low level of fear of movement in the population under study. As most participants were recruited via newspapers or newsletters, it seems reasonable that only patients who did not have fear of activity-induced worsening of symptoms would have applied to participate. We also investigated whether the self-efficacy and control competence subcompetencies of the physical activity–related health competence model improved after taking part in the stand-alone mHealth intervention. However, no differences between the IG and CG were observed. In the study population, both measures that included the subscales showed ceiling effects at baseline. Thus, the possibility of change after the intervention phase was limited.

#### Performance Measures

Strength endurance quantified using the 30-second chair stand test increased by 1.7 repetitions for the IG versus the CG. However, this finding was not statistically significant. Therefore, the results are in contrast to those of a study on the effectiveness of a 6-week internet-based exercise intervention against usual care in patients with knee osteoarthritis reporting a statistically significant between-group effect in favor of the intervention. The participants in the IG improved by an average of 4.5 repetitions. The between-group difference after the intervention was 3.4 repetitions [61].

Change in knee extension strength has been described as a mediator for clinical benefit in patients with knee and hip osteoarthritis [62]. Our results showed no improvement in maximum knee extension strength in the IG. This is in contrast to a meta-analysis including results of 10 studies in which low-intensity resistance training reported short-term ESs with a standardized mean difference of 0.5 when compared to the CG. However, there was also a small group of studies that failed to show significant benefits for strength outcomes. The authors of the meta-analysis [63] hypothesized that one reason for the absence of benefit may be related to the low intensity of these exercise programs as too low intensities cannot trigger sufficient muscle activity to promote neuromotor adaptations and hypertrophy to ultimately generate muscle gains [63]. This could also explain the lack of strength improvements in our study as the analysis of the sensor and app log files after the training sessions revealed only a perceived “slight” to “somewhat exhausting” intensity level, and thus, this tends to fall in the subthreshold exercise dose. In the future, patients should be better educated to enable them to independently adjust their training intensity (eg, by choosing a heavier or lighter exercise variation). At the end of an exercise set, a training-effective exercise load in the range of “strenuous” to “very strenuous” should be achieved.

We did not find consistent superiority of the IG versus the CG for postural control outcomes, and we refrain from discussing this further as measurement instruments, postures, and durations differ across trials and the measures used in our study are not part of the recommended set of performance-based measures to assess physical function in people diagnosed with knee and hip osteoarthritis [64].

#### Adherence

The success of exercise therapy and the associated improvements in pain, physical function, and QoL are highly dependent on the maximization of the adherence to exercise. Therefore, it is recommended to supervise exercise sessions at least in the initial exercise period to enhance adherence before continuing exercise independently [65]. Various factors (eg, motivational level, physical status, personal goals, self-regulation, and several extrinsic factors) can impact exercise adherence [47,66]. To overcome these barriers, especially for nonsupervised training, the field of digital-based exercising may offer a different approach for guidance and motivation as well as an independence from time and location. The particular group of sensor-based apps can additionally supervise the training execution and provide real-time feedback. The impact of supporting features for barrier management in eHealth and mHealth technologies may also be the reason for the high adherence rates of up to 82% to 91% for digital-based home exercise interventions (all sources, not only sensor based) in patients with knee osteoarthritis [67,68]. These numbers correspond to the adherence rate of 92.5% (899/972) in our study and exceed reported rates of 62% to 75% for nondigital home exercise interventions [69,70]. The results of our study only refer to the short term. As adherence to exercise is critical for the long-term benefit of lifestyle interventions [13], future studies may investigate whether better adherence to app-guided interventions can be sustained over a longer period as well.

### Limitations

One limitation of our study is related to potential bias due to missing values of the complete case analyses. According to Jakobsen et al [71], a complete case analysis can be applied up to a threshold of approximately 5% missing values. They further report that missing data can be ignored in the analysis if the impact of missing data on the results is negligible. In our study 9.8% of data were missing but no significant differences were observed for the complete case analyses in comparison to the sensitivity analyses (data not shown) for the KOOS subscales. Another limitation is the lack of blinding of participants because of their obvious group assignment. This may have particularly influenced subjective outcome measures in the CG due to a lack of treatment expectations. A major limitation of the study is related to the sample size of the 2 subgroups, IG A and IG AB. Our study was powered for a joint comparison of IG A and IG AB versus the CG. Therefore, subgroup comparisons of IG A and IG AB versus the CG may lack statistical power and generalizability. Due to the relatively small subgroup sample size, additional data are needed to substantiate or revise the findings of a possible additional treatment effect of wearing a knee brace during exercising.

It should also be mentioned that the study design does not allow for clarification on whether the favorable study results of the IGs were the result of app use or just of the fact that participants exercised more than those in the CG. However, the aim of this trial was not to conduct a comparison of different delivery modes of exercise (eg, supervised in person vs stand-alone app) but to obtain first insights into the efficacy of a sensor-based mHealth intervention in comparison to usual care. Further comparative studies are needed to answer the question of which patients respond best to which type of delivery.

### Conclusions

Individuals with knee osteoarthritis undergoing a 12-week sensor-assisted app-based exercise intervention program with or without an additional knee brace experienced positive treatment effects with medical benefits regarding pain relief and improvements in physical function as well as other osteoarthritis-specific concerns compared to those in the CG. Adherence to the exercise intervention was high, and the mobile app can be classified as a safe intervention, with no SAEs being reported. To overcome limitations in the generalizability of the results because of the rather small sample size and the joint comparison of IG A and IG AB, a well-powered trial on the effectiveness of re.flex versus a CG is currently being conducted.

## Appendix

Multimedia Appendix 1 Re.flex manual.

Multimedia Appendix 2 Subgroup analyses for the Knee Injury and Osteoarthritis Outcome Score.

Multimedia Appendix 3 CONSORT-EHEALTH (Consolidated Standards of Reporting Trials of Electronic and Mobile Health Applications and Online Telehealth) checklist (V 1.6.1).

## Abbreviations

AE: adverse event
ANCOVA: analysis of covariance
CG: control group
CONSORT-EHEALTH: Consolidated Standards of Reporting Trials of Electronic and Mobile Health Applications and Online Telehealth
ES: effect size
HRQoL: health-related quality of life
IG A: intervention group with app-based exercise training
IG AB: intervention group with app-based exercise training in combination with a supportive knee brace
IG: intervention group
KOOS: Knee Injury and Osteoarthritis Outcome Score
MCID: minimal clinically important difference
MCII: minimal clinically important improvement
MCS: Mental Component Score
mHealth: mobile health
NSAID: nonsteroidal anti-inflammatory drug
PCS: Physical Component Score
PROM: patient-reported outcome measure
QoL: quality of life
SAE: serious adverse event
