# Supplementary material for: Efficacy of the mHealth-Based Exercise Intervention re.flex for Patients With Knee Osteoarthritis: Pilot Randomized Controlled Trial
Source: JMIR Mhealth Uhealth. 2024 Sep 9;12:e54356. doi: 10.2196/54356 (PMC11420596; doi:10.2196/54356)
Supplement: Multimedia Appendix 1 [file mhealth_v12i1e54356_app1.pdf]

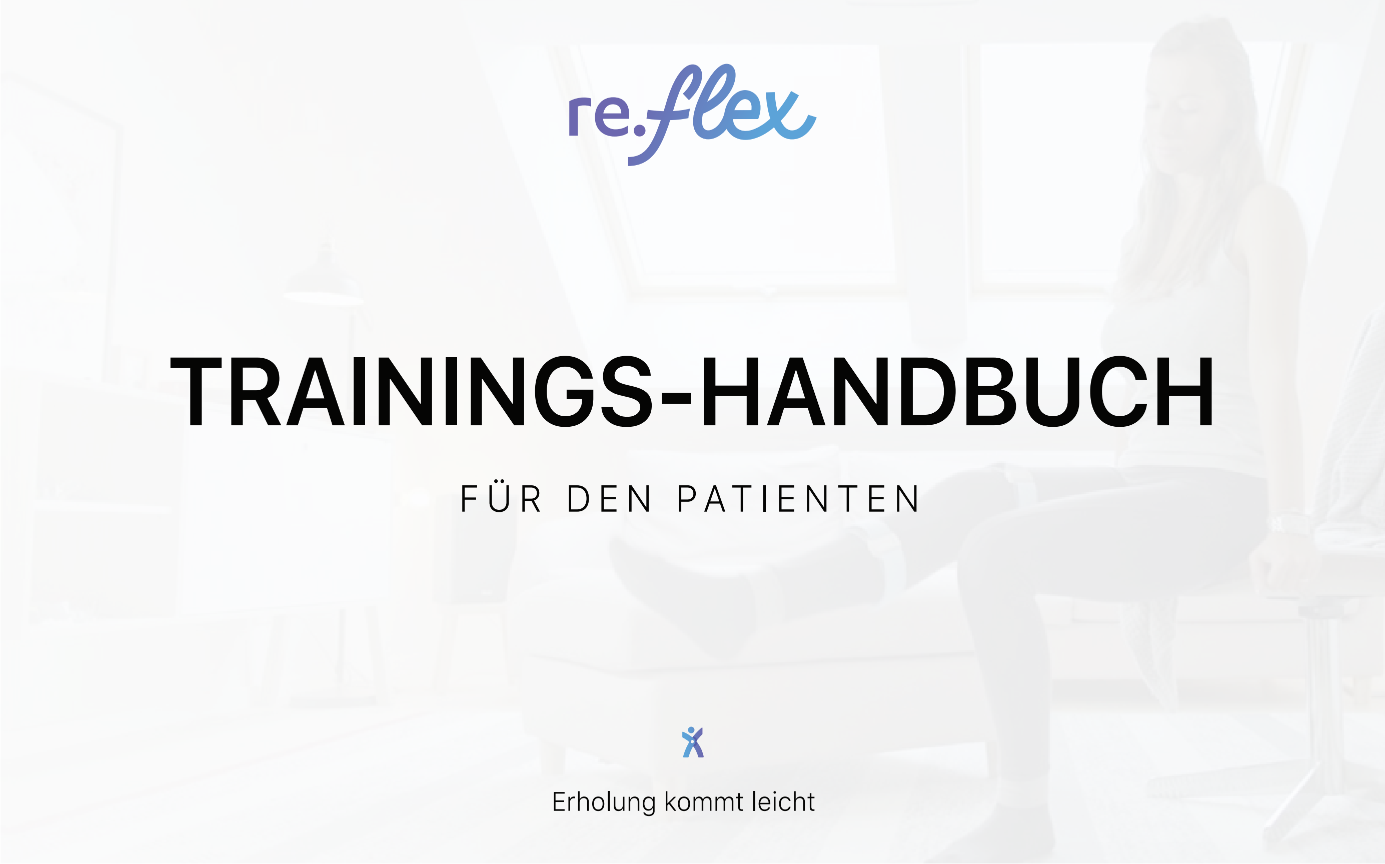

re.flex

TRAININGS-HANDBUCH

FÜR DEN PATIENTEN

Erholung kommt leicht

01

App herunterladen

APP SUCHEN

Öffnen Sie auf Ihrem iPhone den App Store und suchen Sie nach "Reflex Recovery". Drücken Sie auf Laden, um die re.flex App herunterzuladen (Hierfür müssen Sie im WLAN eingeloggt sein). Anschließend können Sie die App in Ihrer Bildschirmübersicht öffnen.

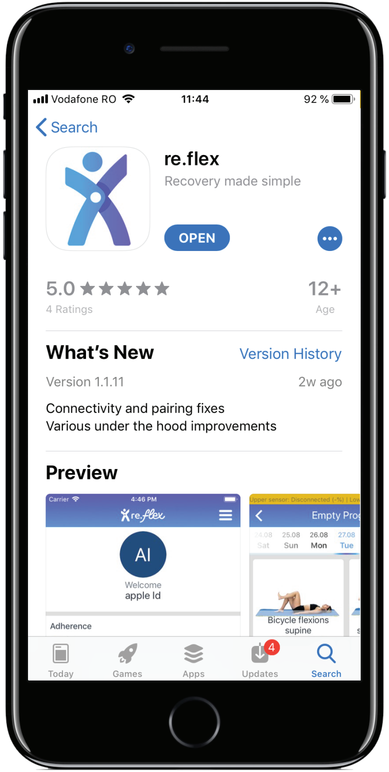

02

Den Sensor laden

Verbinden Sie die Sensoren mit dem Ladekabel. Nach einer Ladezeit von 15 Minuten sind die Sensoren etwa 35% aufgeladen. Werden die Sensoren 60 Minuten geladen, können diese bis zu drei Tage genutzt werden.

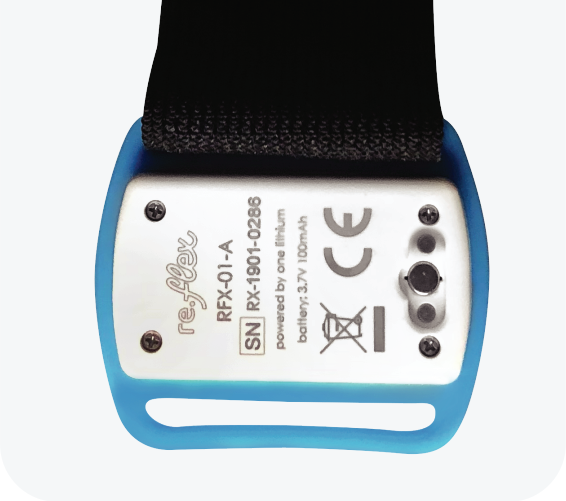

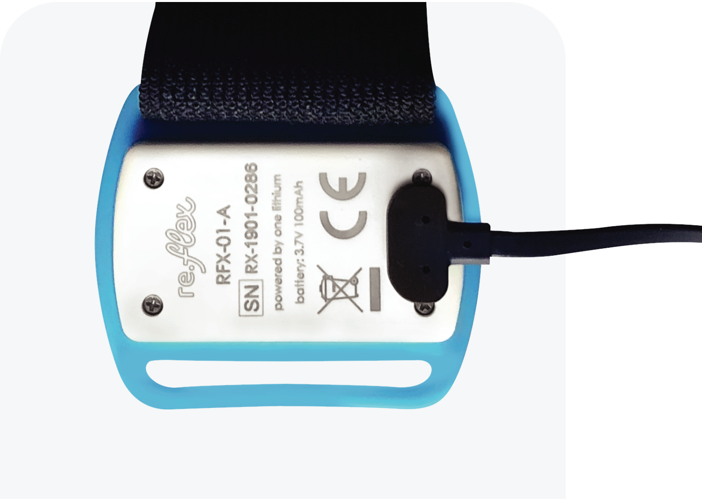

03

Anbringen der Gurte an den Sensoren

Befestigen Sie die Gurte, indem Sie diese durch die Schlaufen der Sensoren führen (wie in der Abbildung gezeigt). Wenn die Gurte angebracht sind und Sie den Buchstaben sehen können, sind die Gurte korrekt ausgerichtet. Abhängig vom Umfang Ihres betroffenen Beins wird der M- oder L-Gurt am Oberschen-kel-Sensor und der S- oder M-Gurt am Unterschenkel-Sensor befestigt.

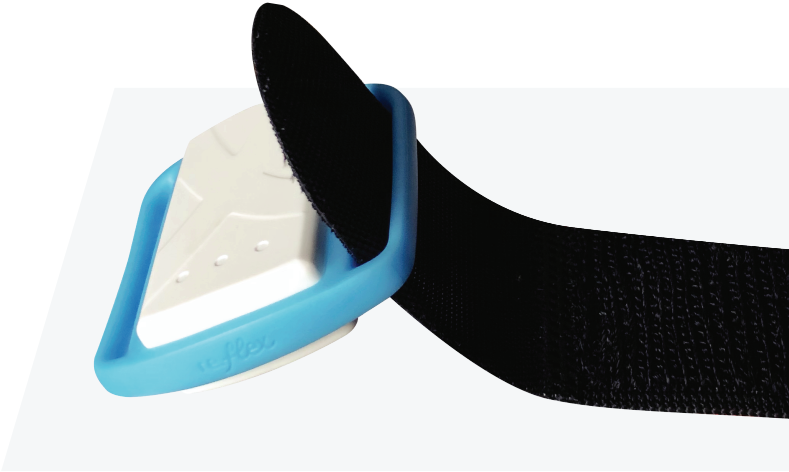

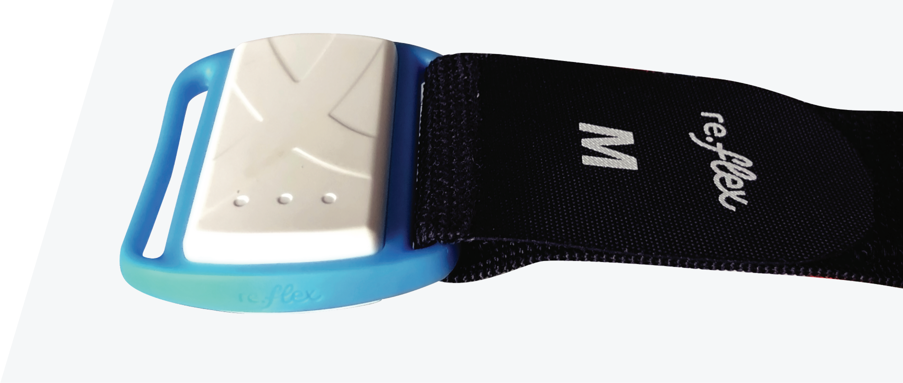

04

Platzieren des Oberschenkel-Sensors

Platzieren Sie einen der Sensoren am betroffenen Bein ca. 10 cm oberhalb der Kniescheibe mittig auf dem Oberschenkel. Achten Sie darauf, die Gurte fest zu schließen, damit die Sensoren nicht verrutschen.

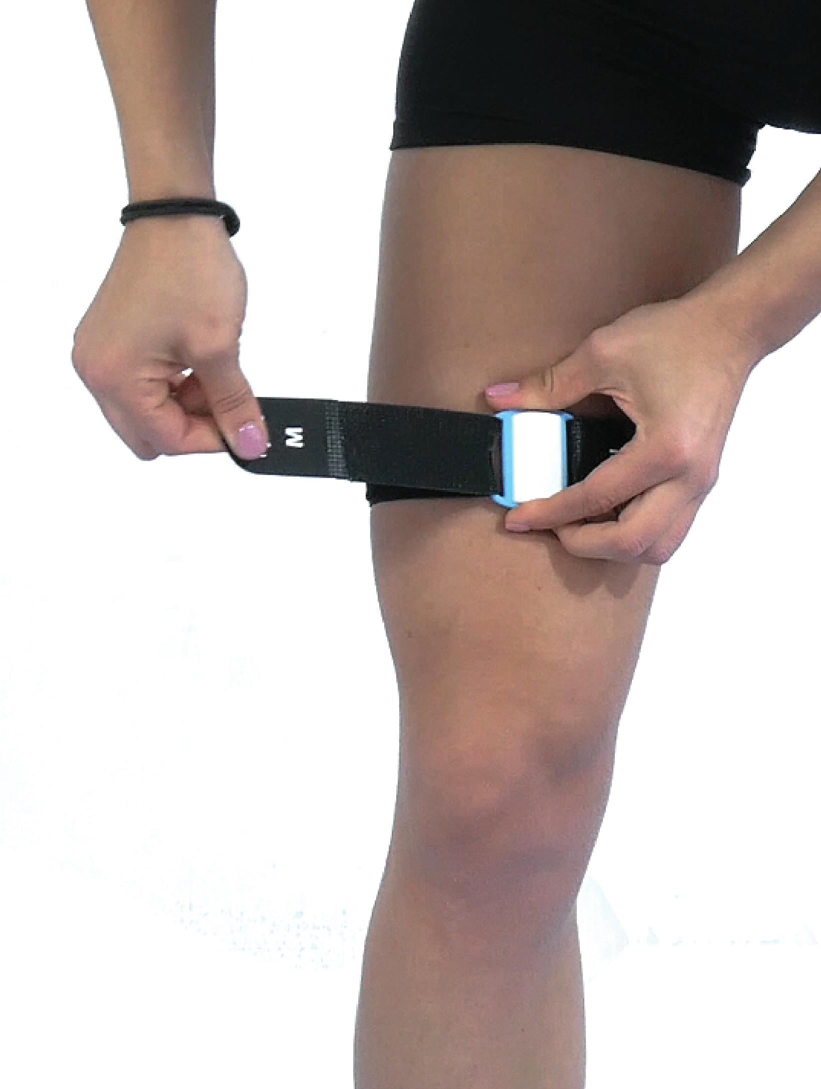

05

Platzieren des Unterschenkel-Sensors

Platzieren Sie den anderen Sensor am betroffenen Bein ca. 10 Zentimeter unterhalb der Kniescheibe mittig auf dem Unterschenkel. Achten Sie darauf, die Gurte fest zu schließen, damit die Sensoren nicht verrutschen.

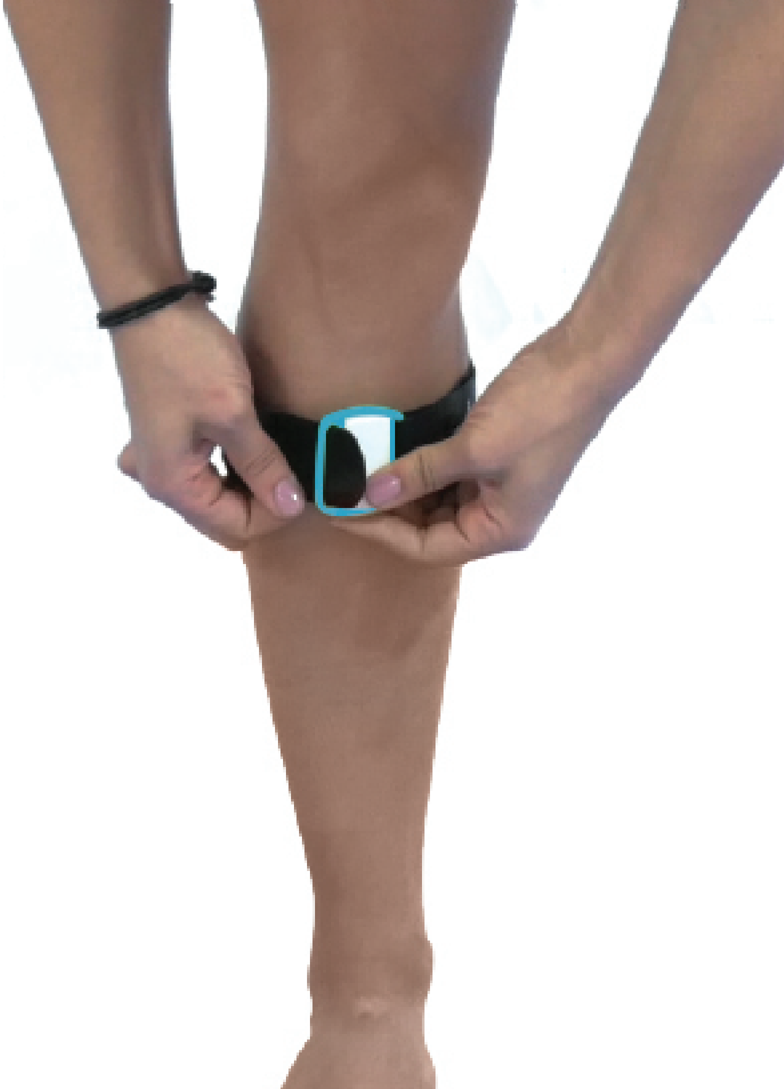

06

Haben Sie eine Knieorthese?

Falls Sie eine Knieorthese verwenden, sind die Sensoren vormontiert. Achten Sie darauf, dass die Sensoren zentriert ausgerichtet sind und fest sitzen, damit sie nicht verrutschen.

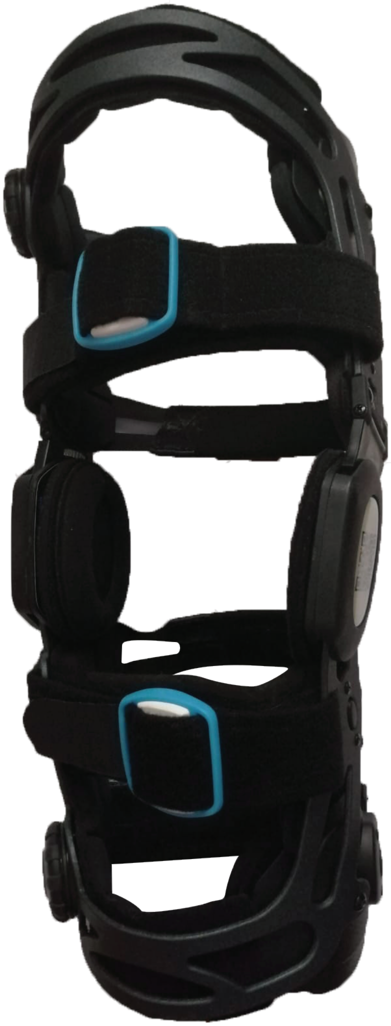

07

Aktive Internetverbindung

Stellen Sie sicher, dass Sie über eine aktive Internetverbindung verfügen, entweder über WLAN oder eine mobile Datenverbindung. Um Ihr WLAN zu aktivieren, wischen Sie mit dem Finger vom unteren Bildschirmrand nach oben und tippen auf das WLAN-Symbol. Alternativ gehen Sie auf Einstellungen -> WLAN und schalten das WLAN ein, um eine Verbindung zu einem bekannten Netzwerk herzustellen.

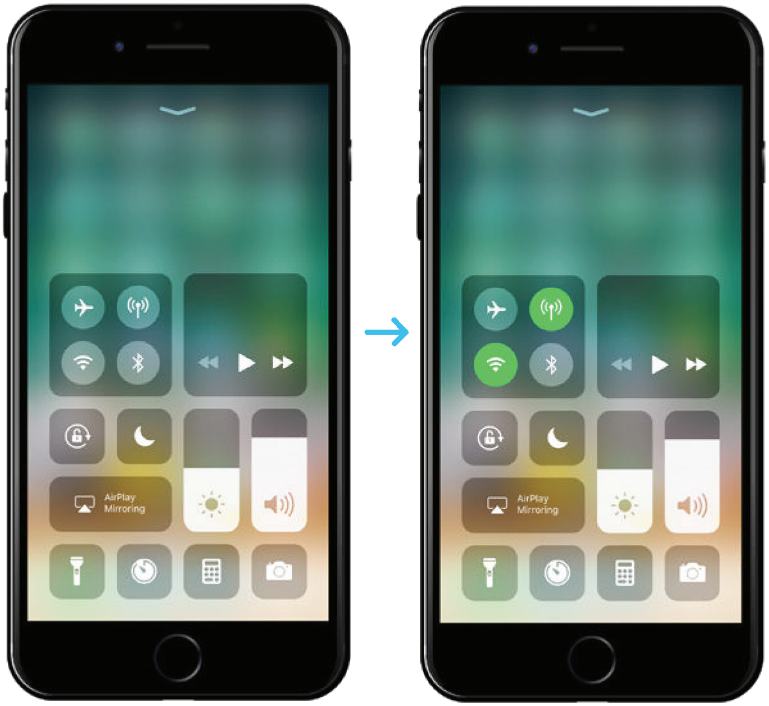

08

Bluetooth anschalten

Wischen Sie mit dem Finger vom unteren Bildschirmrand nach oben und drücken Sie auf das Bluetooth-Symbol

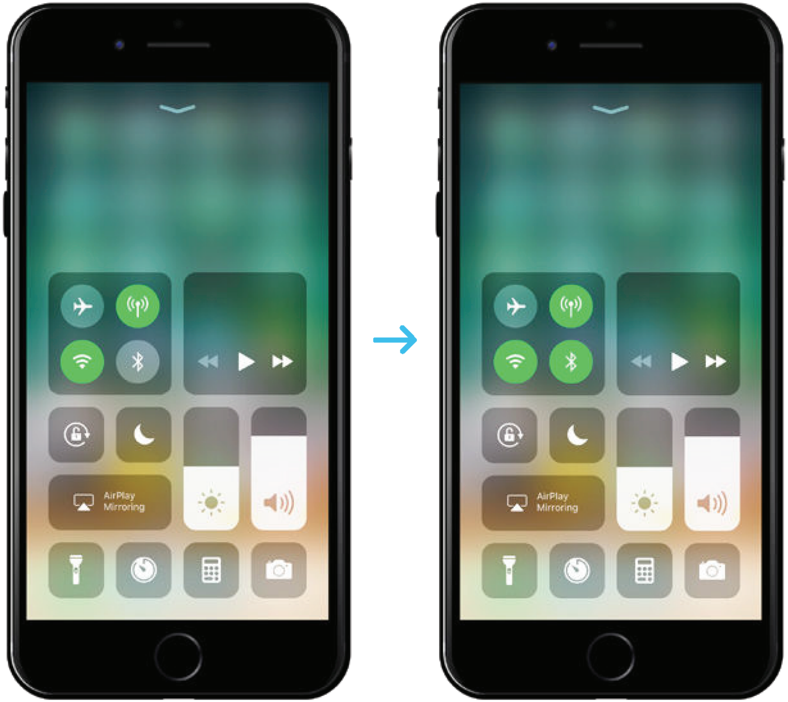

09

App Login

Nachdem Sie die Anwendung gestartet haben, melden Sie sich mit den Login-Daten an, die Sie mit den Sensoren erhalten haben.

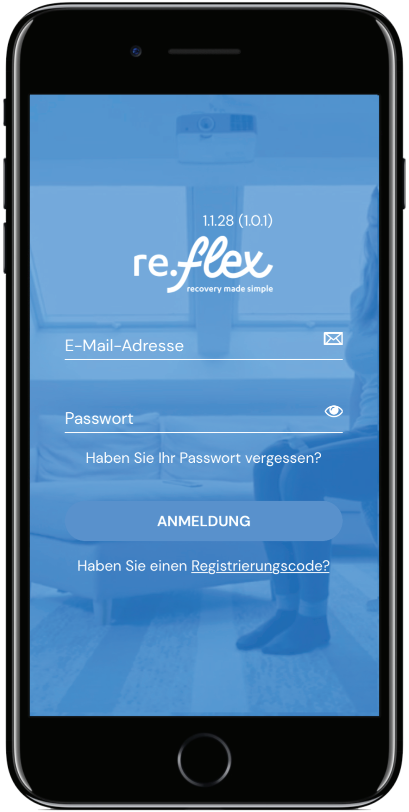

10

Training starten

Wählen Sie “Plan prüfen”. Auf dem folgenden Bildschirm wird eine Vorschau des Trainingsplans angezeigt. Wählen Sie “Training starten”, um mit dem Training zu beginnen.

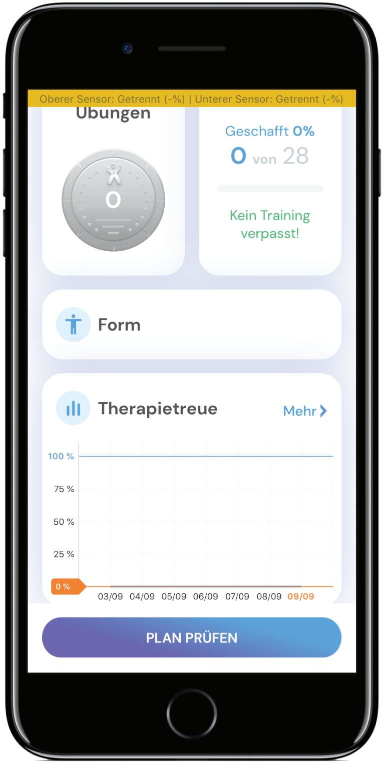

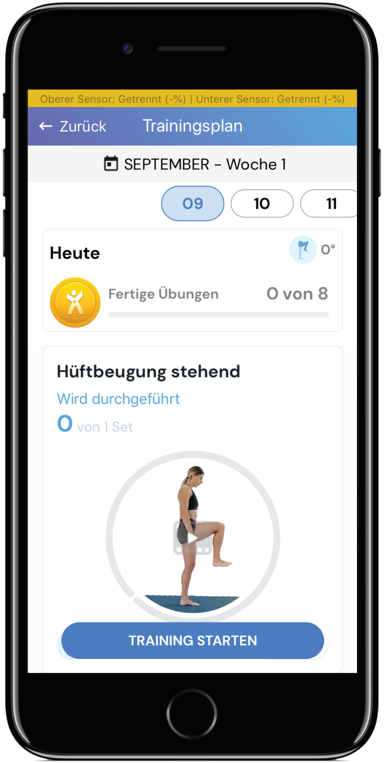

11

Verbinden des Oberschenkel-Sensors

Legen Sie das Mobilgerät auf den Oberschenkel-Sensor und drücken Sie auf dem Mobilgerät die Taste "Verbinden".

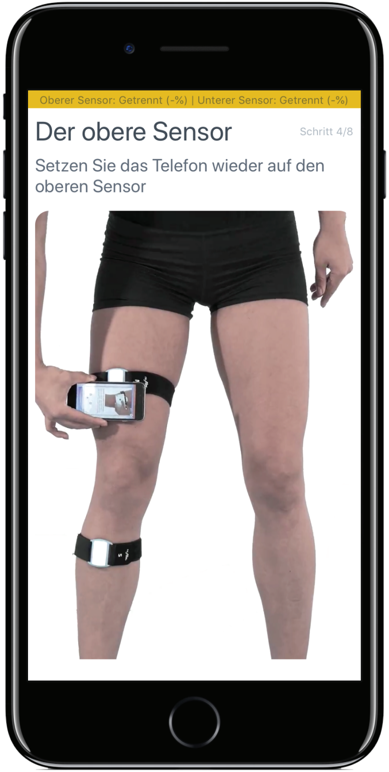

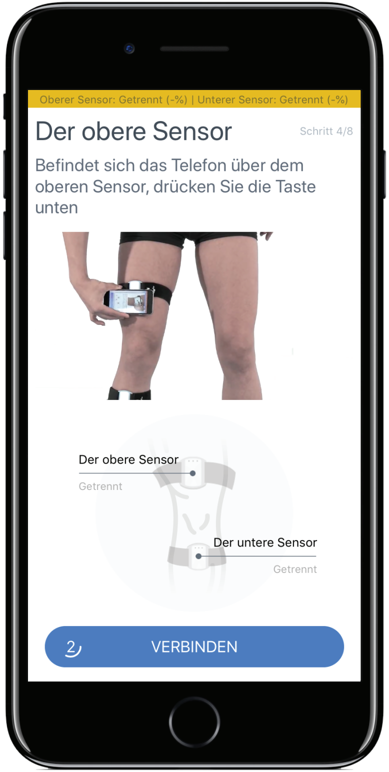

12

Verbinden des Oberschenkel-Sensors

Warten Sie, bis der Countdown abgelaufen ist, während sich das Mobilgerät weiter über dem Oberschenkel-Sensor befindet. Wenn der Sensor zu vibrieren beginnt und blinkt, drücken Sie "Ja", um die Verbindung aufzubauen.

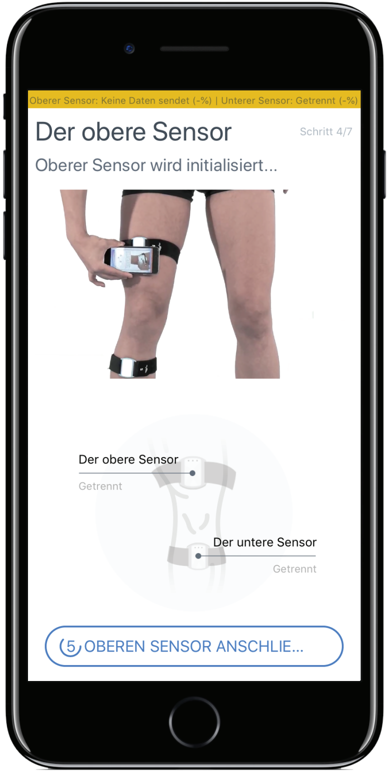

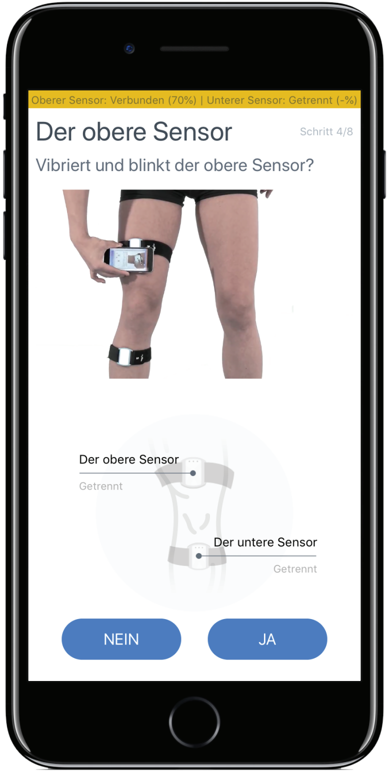

13

Verbinden des Unterschenkel-Sensors

Setzen Sie das Telefon auf den Unterschenkel-Sensor und drücken Sie die Verbindungstaste.

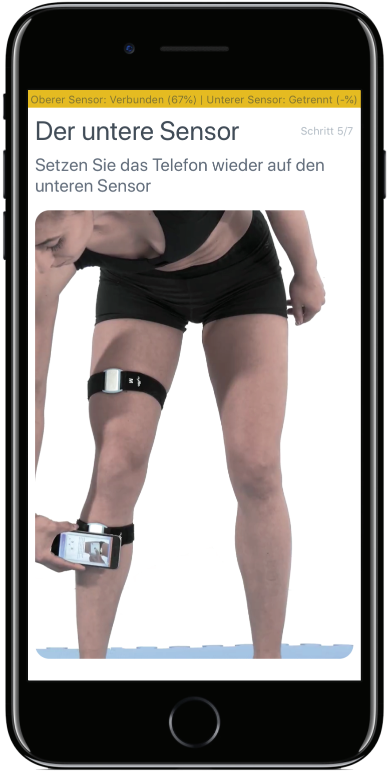

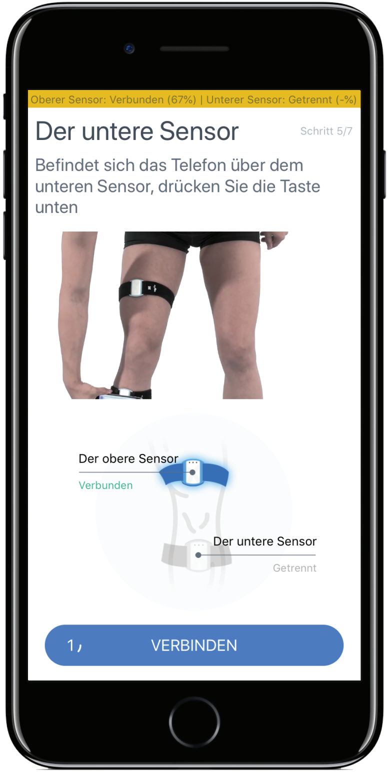

14

Verbinden des Unterschenkel-Sensors

Warten Sie, bis der Countdown abgelaufen ist, während sich das Mobilgerät weiter über dem Unterschenkel-Sensor befindet. Wenn der Sensor zu vibrieren beginnt und blinkt, drücken Sie "Ja", um die Verbindung aufzubauen.

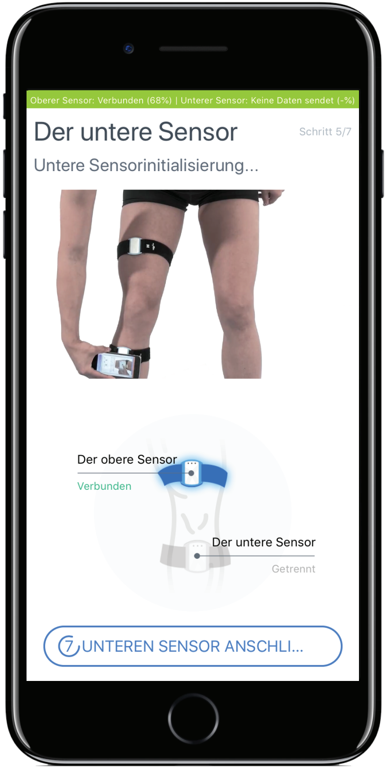

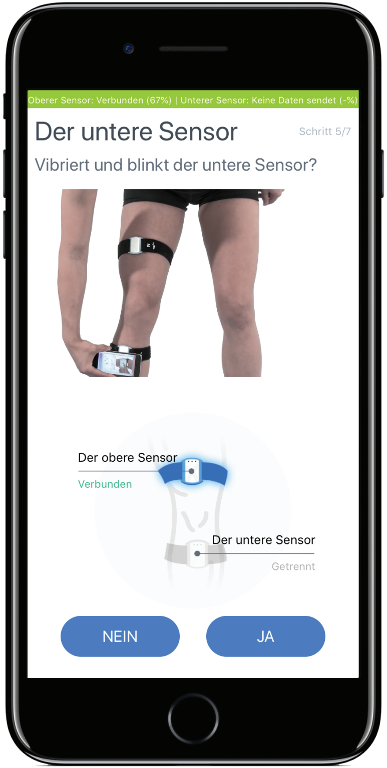

Halten Sie Ihr Knie gestreckt (auf dem Rücken liegend)

1. Kalibrierung in Rückenlage:

Wenn Sie re.flex verwenden, legen Sie sich mit gestreckten Beinen auf den Rücken. Halten Sie dabei die Fersen auf dem Boden. Während Sie diese Position beibehalten, drücken Sie "Fertig" und bereiten Sie sich darauf vor Ihr Knie zu beugen, während Ihre Ferse
über den Boden gleitet.

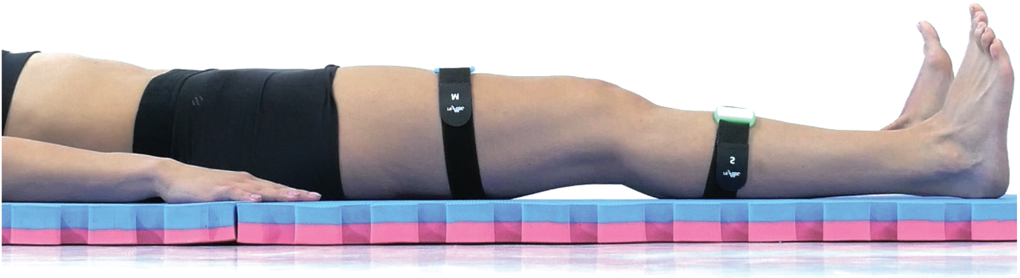

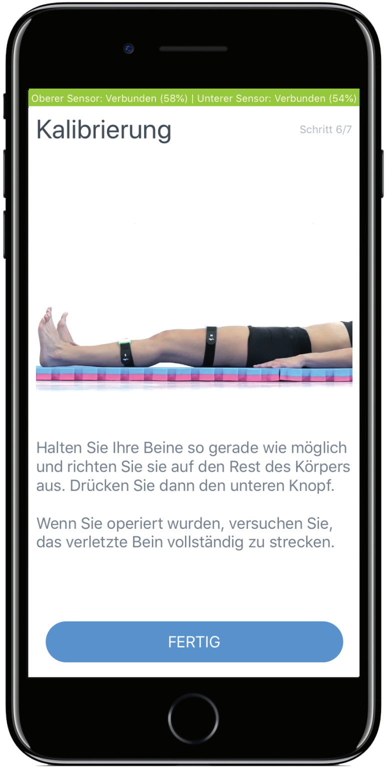

Kalibrierung in Rückenlage

1. Kalibrierung in Rückenlage:

Führen Sie die Kalibrierbewegung aus, in dem Sie Ihr Knie beugen und strecken (bis zur vollständigen Streckung). Dabei gleitet die Ferse über den Boden. Sie müssen diese Bewegung so lange ausführen, bis der Countdown abgelaufen ist.

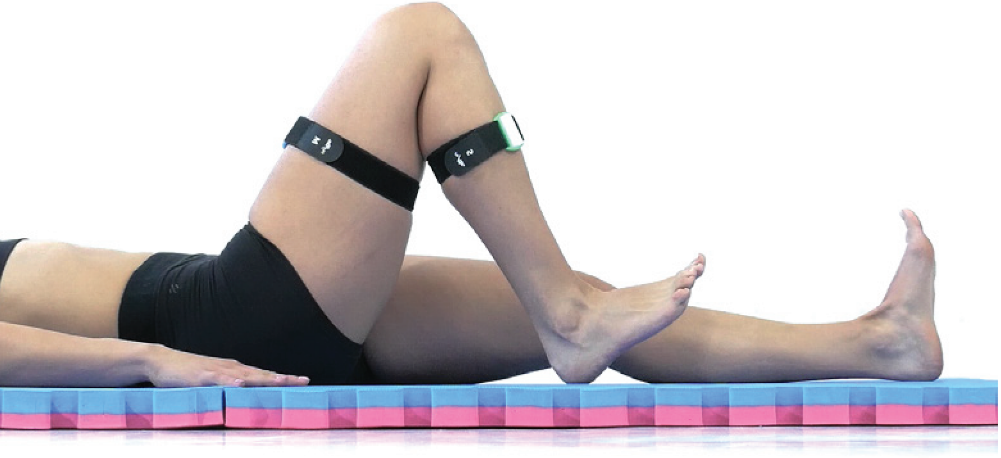

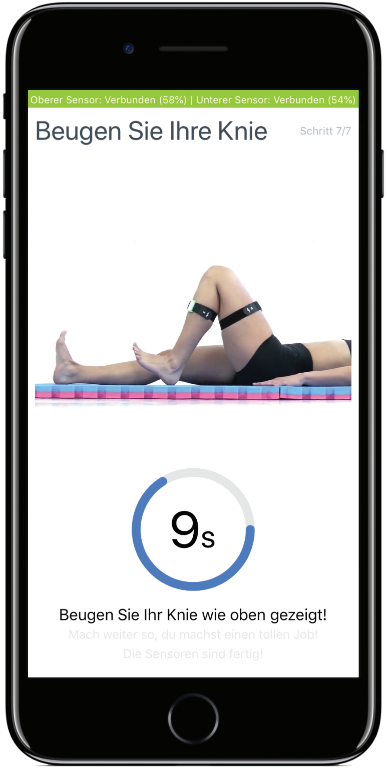

Halten Sie ihr Knie gestreckt (stehend)

2. Kalibrierung im Stehen

Nehmen Sie eine aufrechte Position mit möglichst gestreckten Knien ein. Die Füße sind etwa in hüftbreitem Abstand aufgestellt und die Zehen sind nach vorne gerichtet. Während Sie diese Position beibehalten, drücken Sie "Fertig" und bereiten Sie sich darauf vor, Ihr Knie zu beugen.

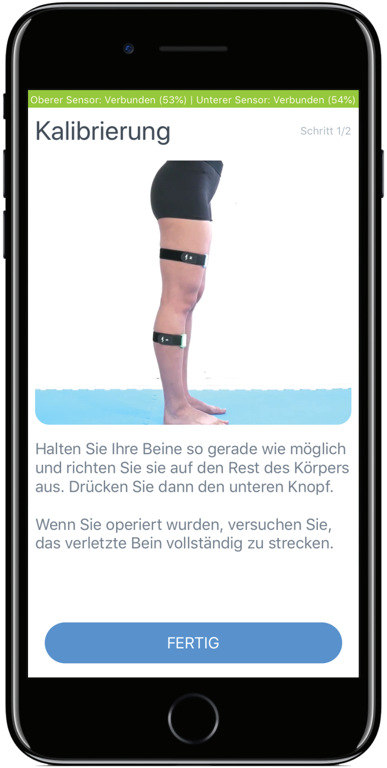

Kalibrierung im Stehen

2. Kalibrierung im Stehen

Bei der Kalibrierung im Stehen führen Sie die Kalibrierbewegung durch, indem Sie das Knie nach oben anheben (siehe Bild) und anschließend mit gestrecktem Knie in die stehende Position zurückkehren. Sie müssen diese Bewegung ausführen, bis der Countdown abgelaufen ist.

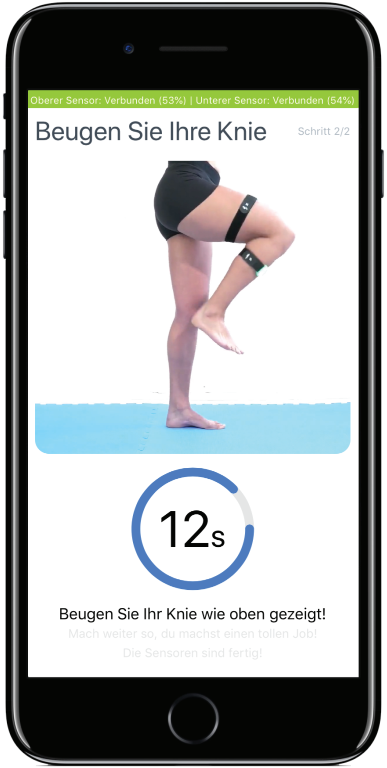

Training

Um eine Übung erfolgreich zu absolvieren, bewegen Sie das Bein wie im Video gezeigt. Ziel ist es die grüne Blase zu füllen (oder zumindest ihren Rand zu berühren). Diese gibt die Bewegungsweite vor. Sollte sich die Blase rot färben, ist die Übungsausführung fehlerhaft. Sie erhalten dann eine Rückmeldung, was Sie bei der nächsten Ausführung ändern sollten. Daraufhin werden Sie aufgefordert in die Ausgangsposition zurückzukehren.

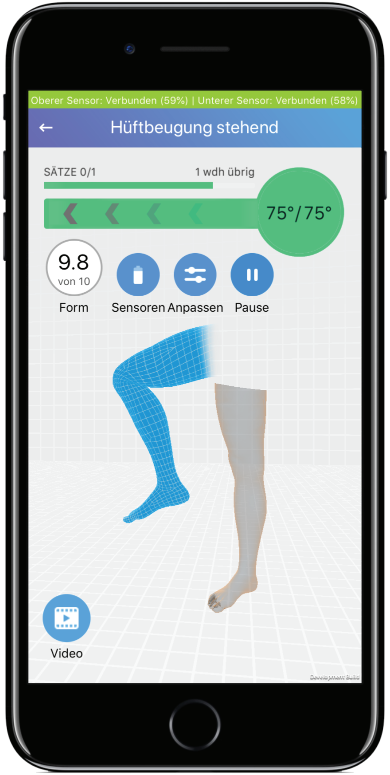

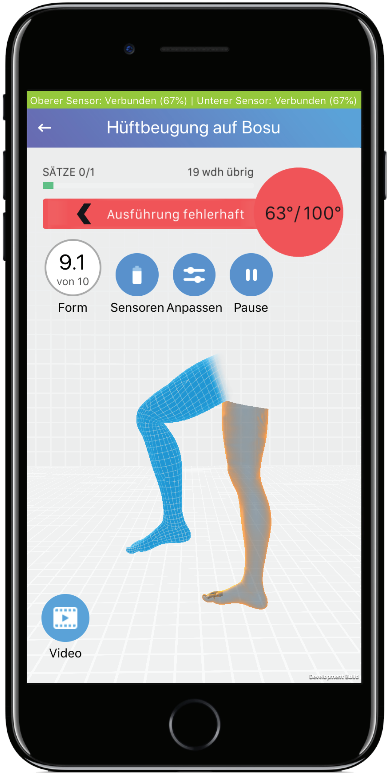

20

Übung wechseln

Drücken Sie in der Trainings-Ansicht den Button “Anpassen” und dann “Übung wechseln”, um die aktuelle Übung mit einer einfacheren oder schwereren Übung auszutauschen.

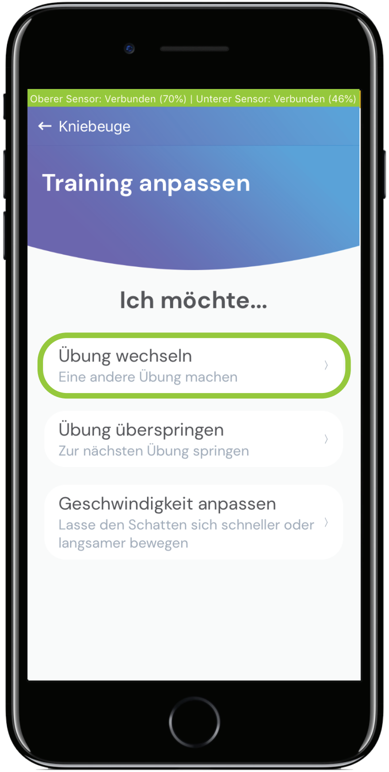

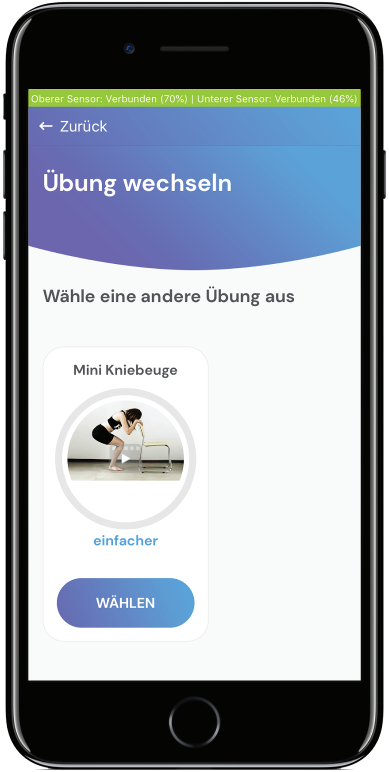

21

Übung überspringen

Drücken Sie in der Trainings-Ansicht den Button “Anpassen” und dann “Übung überspringen”, um die aktuelle Übung zu überspringen. Anwendungsfall: Die Übung ist zu schwer oder aus anderen Gründen nicht machbar.

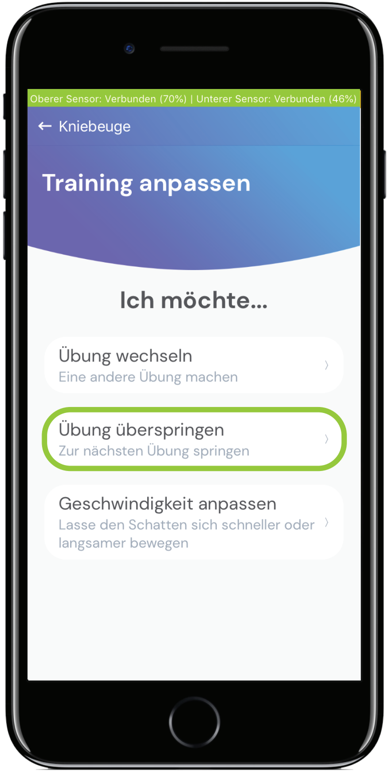

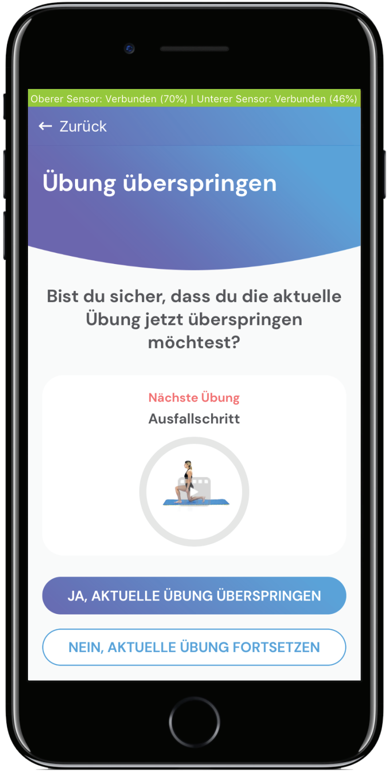

22

Sensoren neu kalibrieren

Drücken Sie in der Trainings-Ansicht den Button “Sensoren” und dann “Neu kalibrieren”, um die Sensoren neu zu kalibrieren. Anwendungsfall: Die Sensoren sind vom Bein gerutscht oder die Ausrichtung des Avatar-Beines auf dem Bildschirm entspricht nicht der Ihres tatsächlichen Beines.

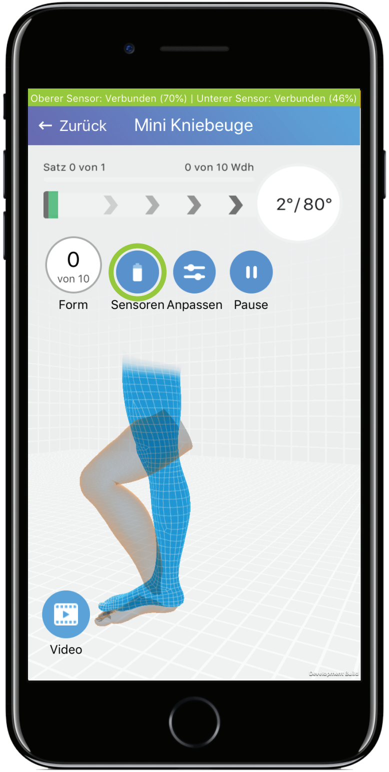

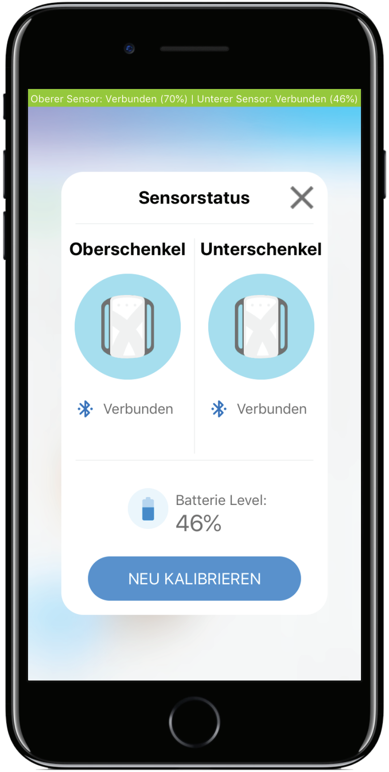

Schmerzbericht und Anstrengun-gsempfinden

Bevor das Training startet, nach jedem Übungssatz sowie am Ende nach dem Training werden Sie gebeten über die empfundenen Schmerzen sowie Ihr Anstrengungsempfinden zu berichten. Drücken Sie dafür einfach auf den Smiley, der am ehesten zu Ihrem Schmerz- und Anstrengungsempfinden passt.

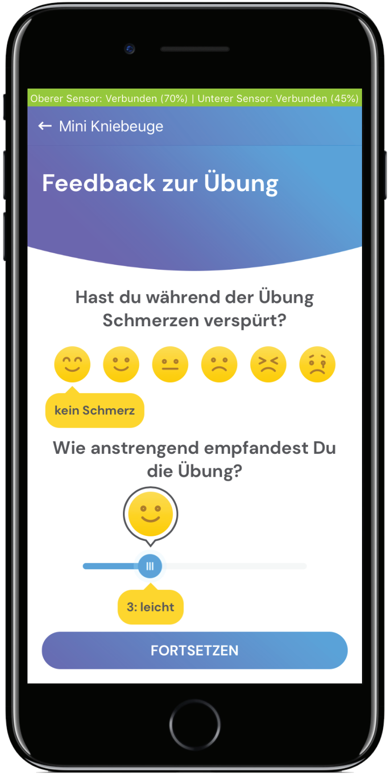

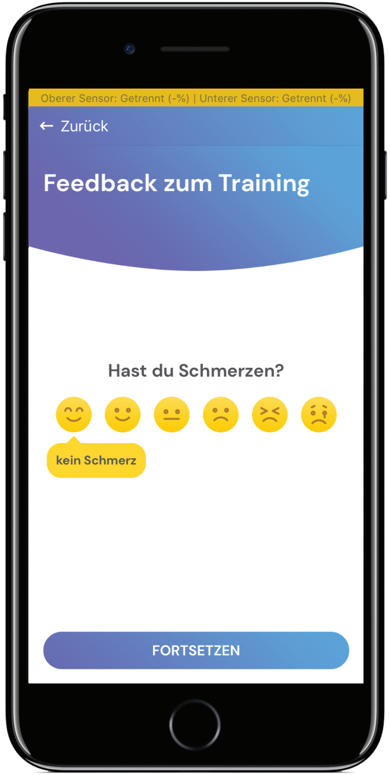

re.flex

Danke, dass Sie sich für re.flex entschieden haben.

Wir wünschen Ihnen eine schnelle Genesung!

Einfach erholen.
