# Supplementary material for: Efficacy of the mHealth-Based Exercise Intervention re.flex for Patients With Knee Osteoarthritis: Pilot Randomized Controlled Trial
Source: JMIR Mhealth Uhealth. 2024 Sep 9;12:e54356. doi: 10.2196/54356 (PMC11420596; doi:10.2196/54356)
Supplement: Multimedia Appendix 2 [file mhealth_v12i1e54356_app2.pdf]

Table A1: Subgroup analyses for the Knee Osteoarthritis Outcome Score (KOOS).

**Knee Osteoarthritis Outcome Score** (KOOS, 0-100 worst to best)	**Group (n)**	**Mean (SE_{mean})** t0	**Mean (SE_{mean})** t3[b]	**Mean difference (95 % CI)**		***P* value**	**ES[a]**
Pain Subscale	IG-A (15)<br>IG-AB (11)	49.8 (3.7)<br>52.5 (5.5)	61.1 (3.3)<br>68.8 (3.8)	IG-A – IG-AB	-7.7 (-18.1 to 2.7)	.139	
	IG-A (15)<br>C (29)	49.8 (3.7)<br>56.2 (2.9)	64.1 (2.4)<br>53.6 (1.7)	IG-A – C	10.5 (4.6 to 16.5)	< .001	0.62
	IG-AB (11)<br>C (29)	52.5 (5.5)<br>56.2 (2.9)	72.2 (3.2)<br>54.8 (2.0)	IG-AB – C	17.4 (9.7 to 25.1)	< .001	1.01
Symptoms Subscale	IG-A (15)<br>IG-AB (11)	51.2 (4.0)<br>58.4 (6.2)	60.5 (3.9)<br>67.1 (4.6)	IG-A – IG-AB	-6.6 (-19.1 to 5.9)	.286	
	IG-A (15)<br>C (29)	51.2 (4.0)<br>58.9 (3.2)	62.3 (3.8)<br>54.8 (2.7)	IG-A – C	7.5 (-2.0 to 16.9)	.117	
	IG-AB (11)<br>C (29)	58.4 (6.2)<br>58.9 (3.2)	70.0 (3.8)<br>56.7 (2.3)	IG-AB – C	13.4 (4.4 to 22.3)	.005	0.73
Physical function (ADL) Subscale	IG-A (15)<br>IG-AB (11)	69.4 (3.8)<br>68.2 (5.2)	76.3 (3.7)<br>80.9 (4.3)	IG-A – IG-AB	-4.7 (-16.3 to 7.0)	.418	
	IG-A (15)<br>C (29)	69.4 (3.8)<br>71.8 (3.5)	78.2 (2.6)<br>67.9 (1.8)	IG-A – C	10.3 (3.9 to 16.7)	.002	0.51
	IG-AB (11)<br>C (29)	68.2 (5.2)<br>71.8 (3.5)	82.4 (3.1)<br>67.9 (1.9)	IG-AB – C	14.5 (7.1 to 21.8)	< .001	0.82
Sport/Recreation Subscale	IG-A (15)<br>IG-AB (11)	35.7 (6.0)<br>31.4 (5.9)	43.2 (5.1)<br>55.2 (6.0)	IG-A – IG-AB	-12.1 (-28.4 to 4.3)	.140	
	IG-A (15)<br>C (29)	35.7 (6.0)<br>33.6 (4.0)	43.2 (3.3)<br>38.0 (2.4)	IG-A – C	5.2 (-3.0 to 13.4)	.210	
	IG-AB (11)<br>C (29)	31.4 (5.9)<br>33.6 (4.0)	54.8 (4.8)<br>37.0 (2.9)	IG-AB – C	17.8 (6.5 to 29.2)	.003	0.82

Table A1: Subgroup analyses for the Knee Osteoarthritis Outcome Score (KOOS) (continued).

Quality of life (QoL) Subscale	IG-A (15)	38.3 (4.0)	43.7 (2.8)	IG-A – IG-AB	-8.7 (-17.5 to 0.2)	.054	
	IG-AB (11)	39.2 (5.1)	52.4 (3.2)				
	IG-A (15)	38.3 (4.0)	43.8 (2.6)	IG-A – C	8.7 (2.2 to 15.1)	.009	0.56
	C (29)	39.2 (2.8)	35.1 (1.9)				
	IG-AB (11)	39.2 (5.1)	52.9 (2.8)	IG-AB – C	17.5 (10.9 to 24.2)	< .001	1.15
	C (29)	39.2 (2.8)	35.3 (1.7)				

t0: Baseline; t3: 12 weeks post baseline
SE_{mean}: standard error of mean; CI: confidence interval
IG-A: intervention group A (only app-assisted exercise training), IG-AB: intervention group AB (app-assisted exercise training in combination with an axis-correcting knee brace), C: control group
[a]ES: effect size, were only calculated for significant results.
[b] Reporting the baseline adjusted means.
